# A new cross-conjugated mesomeric betaine†

**DOI:** 10.1039/d1ra03981d

**Published:** 2021-07-21

**Authors:** Nivedita Sharma, Manjinder Kour, Raakhi Gupta, Raj K. Bansal

**Affiliations:** The IIS (deemed to be University) Jaipur 302020 India bansal56@gmail.com

## Abstract

Cross-conjugated mesomeric betaine (CCMB) has been defined as the dipolar species in which positive and negative charges are *exclusively* restricted to different parts of the molecule. In contrast to indolizine which undergoes [8+2] cycloaddition with dimethyl acetylenedicarboxylate (DMAD), its 1-aza analogue, namely imidazo[1,2-*a*]pyridine reacts with the same reagent to afford the first representative of the CCMB isoconjugate with the odd non-alternant hydrocarbon anion. The structure of the product could be assigned on the basis of the NMR and HRMS results. Furthermore, the spectral studies indicated the presence of additional DMAD molecules in CCMB, possibly in the form of a charge-transfer (CT) complex. The whole sequence of reactions initiated by the attack of imidazo[1,2-*a*]pyridine on DMAD could be rationalized on the basis of the computational study of a model reaction sequence at the DFT (B3LYP/6-31+G(d)) level indicating the formation of a new CCMB derivative. The electronic excited states of the product were investigated by time-dependent density functional theory (TDDFT) calculations at the wB97XD/6-311++G(d,p) level, which indicate low-lying charge transfer that is characteristic of the CCMBs.

## Abstract 2

Cross-conjugated mesomeric betaine (CCMB) has been defined as the dipolar species in which positive and negative charges are exclusively restricted to different parts of the molecule. A new CCMB isoconjugate with odd non-alternant hydrocarbon anion is reported.

## Graphical abstract

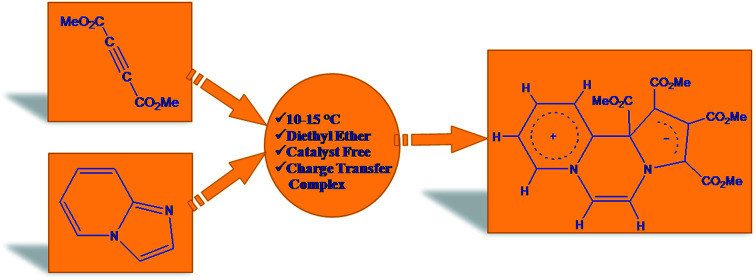

## Introduction

**Scheme 1:**
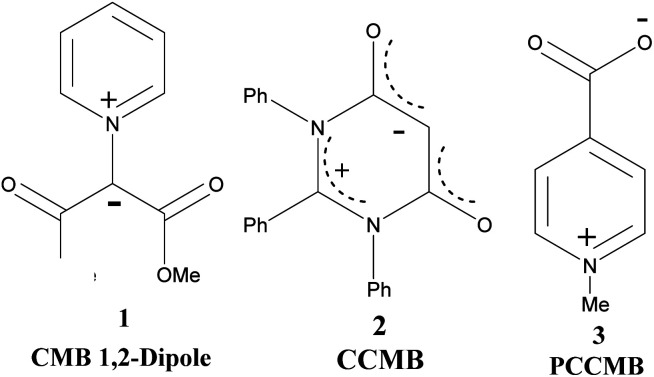
Examples of a CMB (1), CCMB (2) and PCCMB (3)

Ollis, Stanforth and Ramsden defined mesomeric betaines (MB) as the neutral conjugated molecules which can be represented only by dipolar structures in which both positive and negative charges are delocalized within the π-electron system.^1^ They further classified heterocyclic mesomeric betaines into three distinct groups, namely conjugated mesomeric betaines (CMBs), cross-conjugated mesomeric betaines (CCMBs) and pseudo-cross-conjugated mesomeric betaines (PCCMBs) on the basis of the extent of delocalisation of the positive and negative charges. Heterocyclic N-ylides belong to the category of CMBs and can be represented satisfactorily by a 1,2-dipolar structure. The heterocyclic CCMBs are cyclic mesomeric betaines in which the positive and negative charges are *exclusively* restricted to the separate parts of the π-electron systems of the molecule, whereas in the heterocyclic PCCMBs, positive and negative charges are *effectively but not exclusively* restricted to the separate parts of the π-electron systems of the molecule. One example of each of these three categories is shown in Scheme 1.

Potts and co-workers further refined the classification scheme and reported the synthesis and characterization of a large number of CCMB and PCMB systems.^2^ The geometrical data of such systems calculated theoretically were compared with the experimental results. The reactivity was correlated with the frontier molecular orbitals (FMO) computed at the semiempirical level.^3^

In recent years, the CCMBs and PCCMBs have attracted much attention due to their applications in developing new materials used in switchable devices and also in pharmaceuticals. There is a great demand for new switchable materials with high storage capacity of data memory.^4^ A mesoionic group covalently bonded to a polymer affords stable, easy-to-handle, film-forming materials.^5^ In view of this, there have been consistent efforts to synthesise new polymerisable mesoionic heterocyclic compounds.^6,7^ Schmidt *et al.* described the synthesis of polymeric mesomeric betaines by quaternization of poly(4-vinylpyridine) with different halide-containing quinone derivatives and subsequent hydrolysis.^8^ Besides, CCMBs have been used for the synthesis of pharmacologically active molecules. For example, an efficient stereocontrolled route to the isoschizozygane alkaloid core was developed utilizing an intramolecular 1,4-dipolar cycloaddition of a cross-conjugated heteroaromatic betaine.^9^

Annelated pyridine is an important skeleton present in a large number of natural products and pharmacologically active compounds. Pyrrolo[1,2-*a*]pyridine, *i.e.* indolizine and imidazo[1,2-*a*]pyridine are two such structural scaffolds which constitute various biologically active molecules.^10,11^ In view of this, chemists and pharmacologists make consistent and continuous efforts to enlarge the libraries of these structural motifs and develop new protocols. Cycloadditions^12^ and Michael reactions^13^ are two important synthetic strategies that have been often employed with much success for making new C–C and C–N and other carbon-heteroatom bonds.

**Scheme 2:**
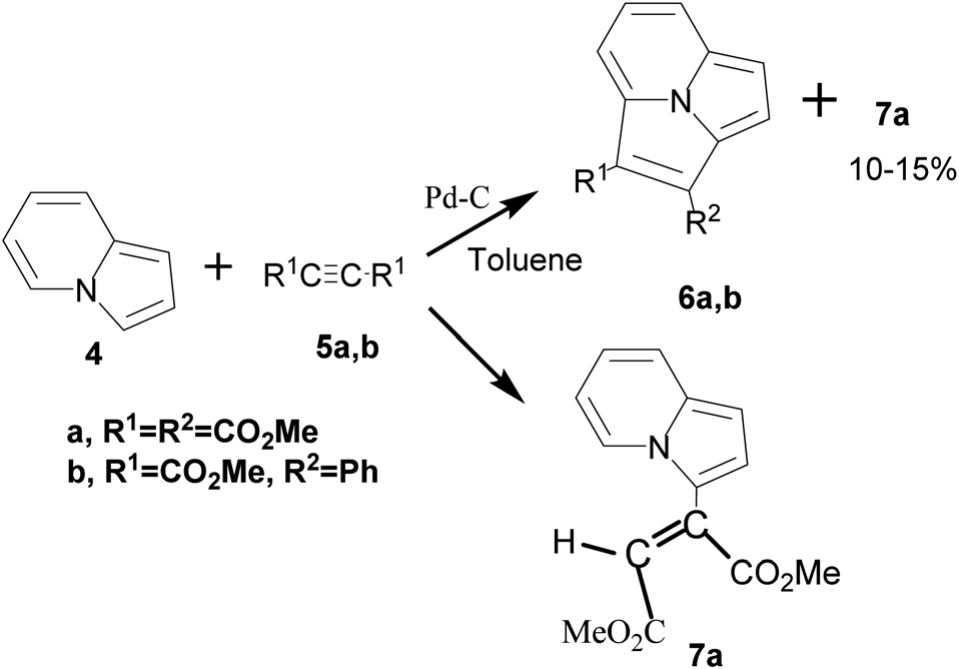
Reaction of indolizine with acetylenecarboxylic acid derivatives.

Boekelheide and co-workers for the first time studied the reaction of indolizine with acetylenecarboxylic acid derivatives in the presence/absence of the dehydrogenating catalyst, palladized charcoal.^14^ In the presence of the catalyst, [8+2] cycloadduct 6 was formed as the main product, whereas in the absence of the catalyst, the reaction stopped at the stage of the formation of the Michael adduct 7 (Scheme 2).

**Scheme 3:**
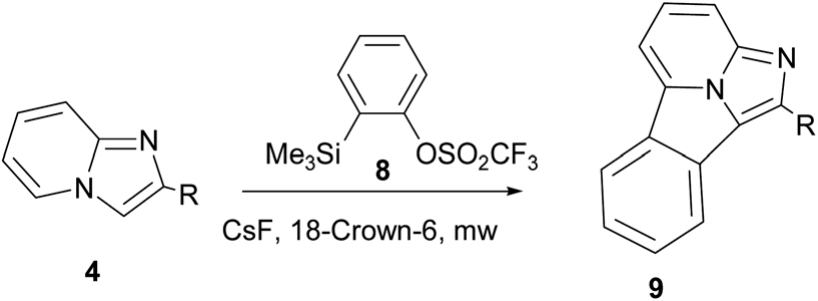
[8+2] Cycloaddition of 2-substituted imidazo[1,2-*a*]pyridines with benzyne generated *in situ*.

Cossio and co-workers reported [8+2] cycloaddition of 2-substituted imidazo[1,2-*a*]pyridines (4) with benzyne generated *in situ* from 2-(trimethylsilyl)phenyl trifluoromethylsulphonate (8) under microwave irradiation (Scheme 3).^15^

On the basis of the DFT calculations, a highly asynchronous concerted [π8s+π2s] mechanism was established.^15^

With this background, we were motivated to study the reaction of imidazo[1,2-*a*]pyridine with DMAD as no literature report could be found in this regard. On adding a solution of DMAD to imidazo[1,2-*a*]pyridine solution, an exothermic cascade reaction ensued and the product was identified as the first representative of the CCMB isoconjugate with the odd non-alternant hydrocarbon anion; the experimental and theoretical results are presented here.

## Results and discussion

**Scheme 4:**
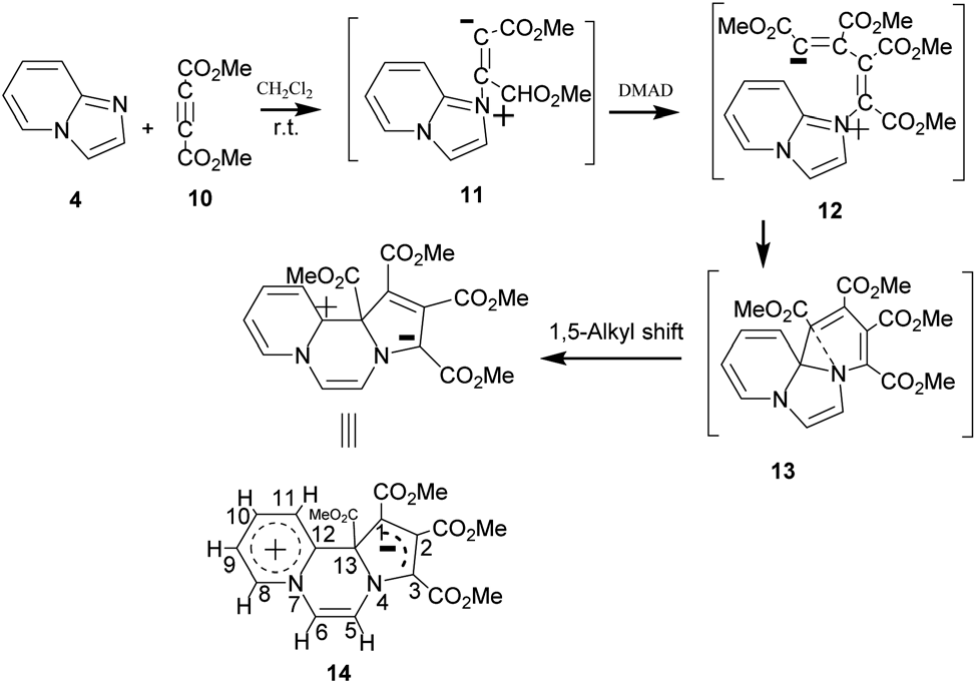
Reaction of imidazo[1,2-*a*]pyridine with DMAD.

On adding DMAD (2 equiv.) into a solution of imidazo[1,2-*a*]pyridine in diethyl ether at low temperature (*ca.* 10–15 °C) under an inert atmosphere, a cascade reaction ensued and a dark brown solid separated (Scheme 4).

The initially formed 1,4-dipole (11) from the reaction of imidazo[1,2-*a*]pyridine (4) with DMAD reacts with a second molecule of DMAD followed by intramolecular cyclisation of the resulting betaine (12) to generate 13. The latter undergoes 1,5-alkyl shift to afford the final product 14.

**Scheme 5:**
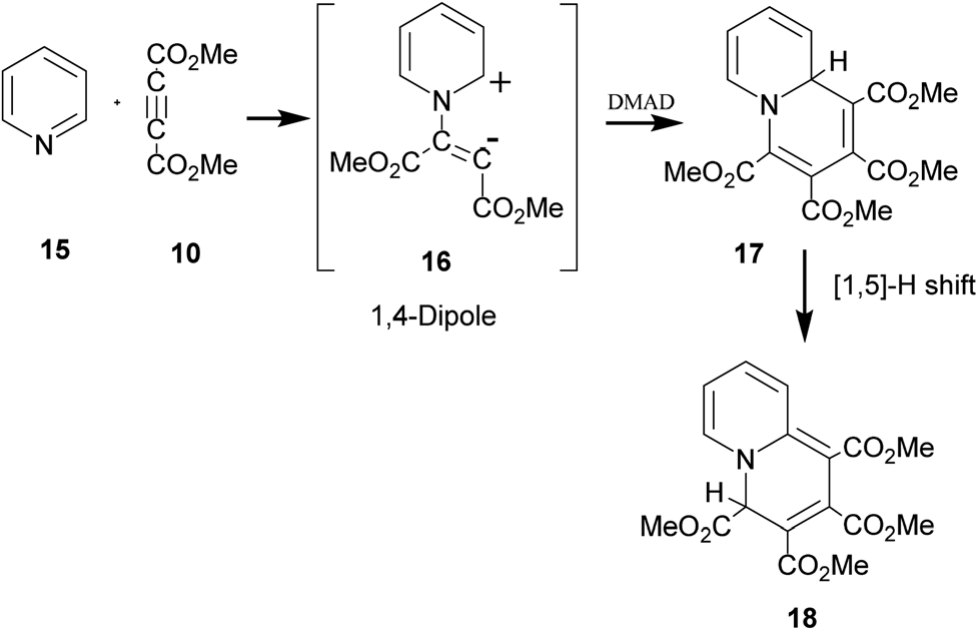
Generation of 1,4-dipole from the reaction of pyridine with DMAD and its subsequent 1,4-dipolar cycloaddition with DMAD.

The above sequence of reactions is analogous to the reaction of pyridine with DMAD first reported by Diels and Alder in 1932 to give two products.^16^ However, the correct structure of the yellow stable compound as tetramethyl 4*H*-quinolizine-1,2,3,4-tetracarboxylate 18 could be established almost three decades later by Acheson and co-workers.^17^ Kamienska-Trela *et al.* managed finally to synthesise specifically the unstable red product of the reaction, which necessitated to follow a special protocol and identified it as tetramethyl 9*aH*-quinolizine 1,2,3,4 tetracarboxylate, the higher energy tautomer 17.^18^ Huisgen and co-workers^19^ rationalised the whole sequence of reactions by postulating the initial formation of a 1,4-dipole 16 from the reaction of pyridine with DMAD, followed by its reaction with a second molecule of DMAD and subsequent [1,5]-H shift to afford the cycloadduct 18 (Scheme 5).

It was termed as 1,4-dipolar cycloaddition, an example of [π4s+π2s] cycloaddition.^19^ In this context, it was emphasized that unlike 1,3-dipoles, 1,4-dipole was not isolable and could be generated *in situ* only.^19^ A variety of imines including aromatic N-heterocycles have been used for generating 1,4-dipoles.^20^ We recently reported *in situ* generation of non-aromatic cycloimines from diazotisation/dediazotisation of *N*-amino cyclic amines followed by their trapping with DMAD to yield tetramethyl 9*H*-5,6,7,8-tetrahydroquinolizine-1,2,3,4-tetracarboxylate and similar products. Experimental results could be rationalised with theoretical calculations.^21^

The structure of the product 14 was established on the basis of the IR and ^1^H NMR data. In the IR spectrum, intense absorption bands at 1725 cm^−1^ (C=O st.) and 1257 cm^−1^ (C(O)–O st.) confirm the ester groups. In the ^1^H NMR spectrum, downfield doublets at *δ* 8.11 (^3^*J*_HH_ = 6.4 Hz) and *δ* 7.61 (^3^*J*_HH_ = 6.8 Hz) result due to H8 and H11 protons respectively. Two triplets at *δ* 7.55 (^3^*J*_HH_ = 6.8 Hz) and *δ* 6.77 (^3^*J*_HH_ = 6.8 Hz) can be assigned to the protons H10 and H9 respectively. The H6 and H5 give partially overlapping doublets at *δ* 7.15 (^3^*J*_HH_ = 5.6 Hz) and *δ* 7.13 (^3^*J*_HH_ = 5.6 Hz) respectively. The methoxy protons give singlets at *δ* 3.71, 3.70, 3.66 and 3.65. As discussed later, there are more singlets at *δ* 3.85, 3.82, 3.77, 3.72 ppm possibly due to two molecules of DMAD present in the form of the CT complex.

### HRMS

In the HRMS, three major peaks are observed at *m*/*z* 403.116 u, 687.192 u and 705.204 u (base peak). The peak obtained at 403.116 u is in perfect agreement with the expected sum formula of the product [calcd for C_19_H_19_N_2_O_8_ 403.114] and is accompanied also by the corresponding isotopic pattern. Two further major peaks at *m*/*z* values of 687.192 u [calcd for C_31_H_31_N_2_O_16_ 687.167] and 705.204 u [calcd for C_31_H_33_N_2_O_17_ 705.178] could be potentially correlated as follows:
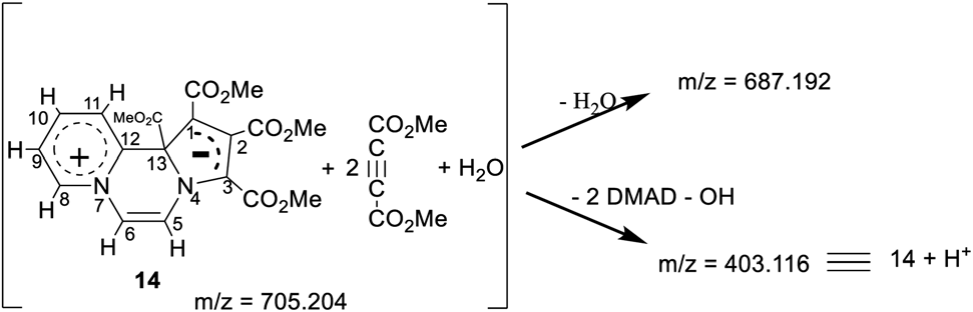


Thus, it appears that the initially formed product 14 takes up two more molecules of DMAD and a molecule of water. It rationalises the presence of a broad absorption band at 3500 cm^−1^ (O–H str.) in the IR spectrum and four additional signals at *δ* 3.85, 3.82, 3.77, 3.72 ppm in the ^1^H NMR spectrum as mentioned earlier. In the absence of an X-ray crystal structure analysis, it is difficult to perceive the pattern of bonding of these two additional molecules of DMAD to 14, *i.e.* whether they are covalently bonded or present as the CT complex. Unfortunately, all our attempts to grow a single crystal proved futile.

A close look at the structure of 14 reveals it to belong to the category of the CCMBs isoconjugate with odd non-alternant hydrocarbon anion.^1^ It appears to be the first representative of this class of mesomeric betaines, as to the best of our knowledge, no compound of this category has been reported so far. In order to explain structural features of this class of CCMBs, some hypothetical examples were included in the review.^1^ In accordance with the structural features of the CCMB mentioned earlier, in 14, positive and negative charges are exclusively restricted to the pyridinium and pyrrole rings respectively, and these two parts are intervened by the pyrazine ring and it is a 1,4-dipole. Furthermore, in consonance with the molecular orbital approach, the delocalised negative charge in 14 is associated with the five-membered ring which is isoconjugate with the odd non-alternant hydrocarbon anion.^1^

### Frontier molecular orbitals

**Fig. 1:**
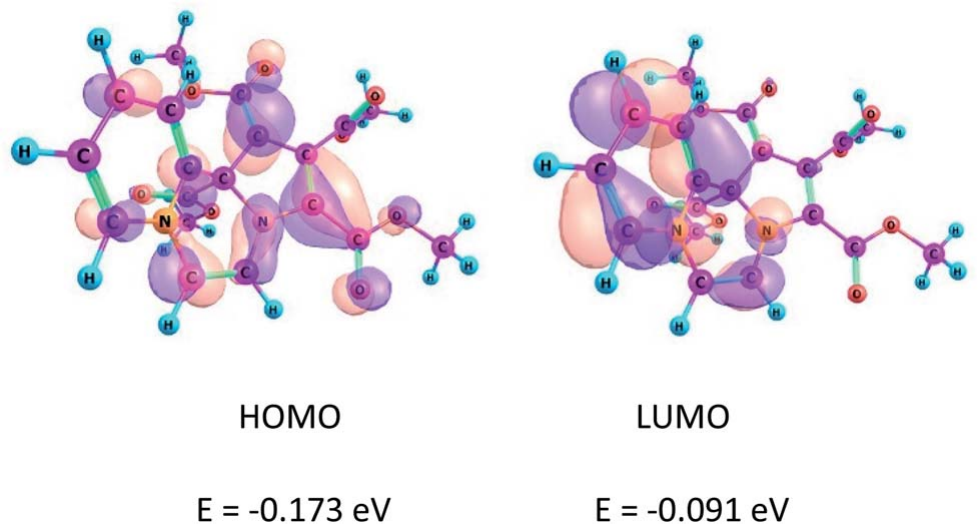
Kohn–Sham FMOs of the compound 14 computed at the B3LYP/6-31+G(d) level.

The location and separation of the HOMO and LUMO in the molecule are other characteristic features of a CCMB.^3,22^ The HOMO and LUMO of the product 14 are shown in Fig. 1.

It may be noted that HOMO is centred on the five-membered ring having the negative charge density, whereas LUMO is located on the six-membered ring, *i.e.* the pyridinium ring. These FMOs are separated by a nodal plane which bisects the middle ring, *i.e.* the pyrazine ring. This feature, namely the separation of the FMOs is in accordance with the CCMB system.

### Spectrophotometric and theoretical studies

In contrast to the CMB species, which acts as 1,3-dipole and undergoes 1,3-dipolar cycloaddition, CCMBs have been reported to react as 1,4-dipoles with electron-deficient dipolarophiles.^1^ In the present case, 1,4-dipolar cycloaddition of 14 with another molecule of DMAD could be ruled out on two counts. Firstly, two ends of the 1,4-dipole present in 14 (C3 and C11) are quite separated (distance 3.5 Å as calculated at the B3LYP/6-31+G(d) level). Secondly, as revealed by Natural Bond Orbital (NBO) analysis, the lone pair of the adjacent N atom is shared with C11 conferring aromaticity on the pyridinium ring. A 1,4-cycloadduct involving C3 and C11 positions will be at the cost of the aromaticity of the pyridinium ring and it is expected to be a highly strained molecule. Nevertheless, as mentioned earlier, additional signals at *δ* ∼ 3.8 indicate presence of additional molecules of DMAD which could not be removed even after repeated maceration of the sample with diethyl ether. Furthermore, a shoulder at 340 nm was also observed in the electronic spectrum of the product. These results indicated the presence of additional DMAD in the form of the CT complex.

In view of this, we investigated CT complex formation between imidazo[1,2-*a*]pyridine and DMAD systematically with UV-visible spectroscopy.

**Fig. 2:**
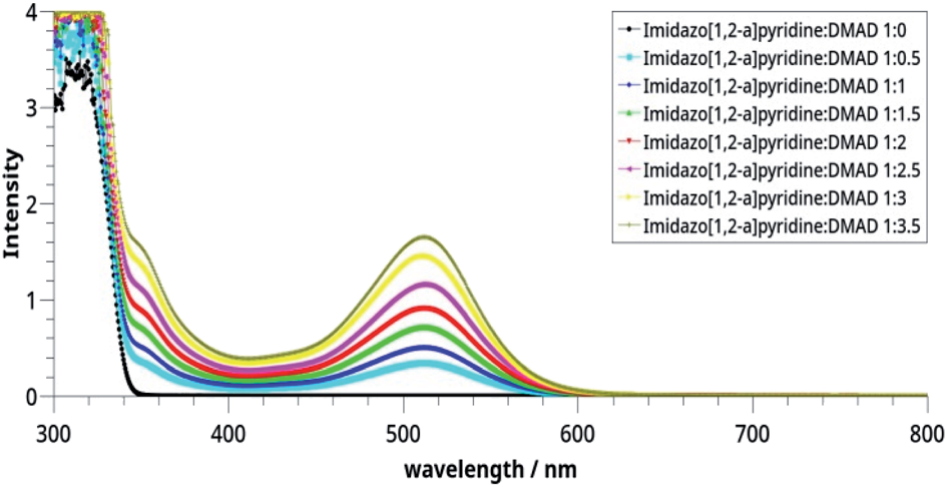
Electronic spectra of the CT complexes between imidazo[1,2-*a*]pyridine and DMAD solutions taken in different concentrations.

**Fig. 3:**
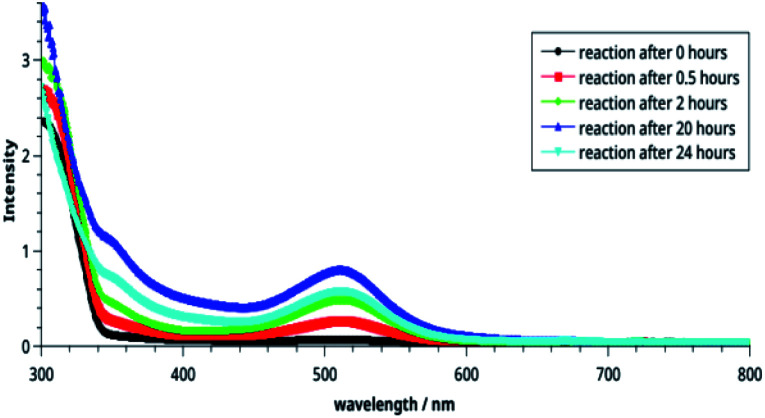
Time-dependent electronic spectra of the CT complexes.

Spectrophotometric^23,24^ and theoretical^25^ studies of the CT complex formed by pyridine derivatives have been reported earlier. For studying CT complex of imidazo[1,2-*a*]pyridine with DMAD, we selected two variables, namely relative concentrations of the donor (imidazo[1,2-*a*]pyridine) and acceptor (DMAD) (Table 2) and time by taking 1 : 1 concentrations of the two reactants. The graphs so obtained are shown in Fig. 2 and 3 respectively.

It may be noted in Fig. 2 that on increasing the concentration of DMAD, intensity of the absorption band at 518 nm increases and becomes maximum with concentration ratio 1 : 3.5. However, change in the intensity while going from 1 : 3 (yellow line) to 1 : 3.5 (grey line) has not been observed as much as it is in the previous step. Intensity of the shoulder at 346 nm follows almost similar trend.

The effect of time on the stability of 14 is illustrated in Fig. 3. It is noteworthy that reaction starts immediately and humps at both wavelengths can be seen after 30 min. The intensities of these bands increase with passage of time and become maximum after 20 h (blue line). Thereafter, it decreases indicating decomposition of the compound 14. From this, it may be concluded that the compound is stable in solution at r. t. for about 20 h.

### Frontier molecular orbital energies of the CCMB

The energy gap between the FMOs, namely HOMO and LUMO is an important parameter for ascertaining the efficacy of an organic photosensitiser; a smaller energy gap reveals its greater efficiency. The cyclic voltammetry (CV) has been often used for determining energies of the FMOs.^26–29^ Non-negligible inaccuracies have been however observed often, which were attributed to the secondary factors, such as atypically dense or loose packing of the molecules in the solid phase and solute–solvent interactions.^30^ In an interesting study, energies of the FMOs determined experimentally using UV-vis spectroscopy and CV were compared with those computed at the DFT level and the latter were found to be in good agreement with the former.^31^ Encouraged by these findings, we too calculated the energies of the FMOs of the CCMB 14 and the reactants at the DFT (B3LYP/6-31+G(d)) level.

**Fig. 4:**
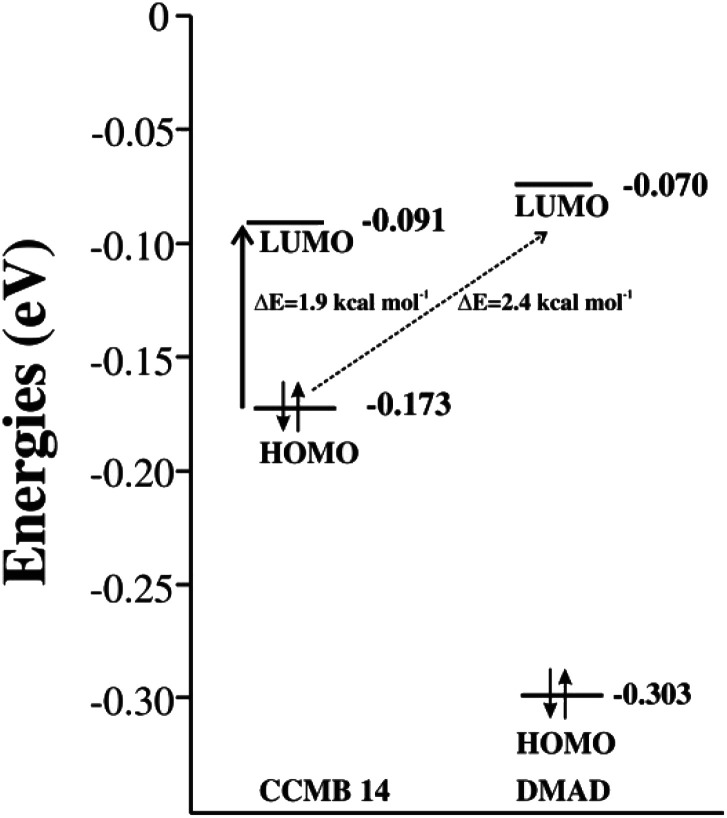
FMOs of the generated CCMB and DMAD.

The intramolecular charge transfer in the CCMB 14 as well as intermolecular charge transfer from the HOMO of the CCMB 14 to the LUMO of DMAD are shown in Fig. 4.

It is noteworthy that the energy gap between the HOMO and LUMO of 14 is 1.9 kcal mol^−1^ only indicating the possibility of an effective intramolecular charge transfer. It is in accordance with the intensity of the absorption band at 518 nm discussed earlier. Furthermore, the energy gap between HOMO of 14 and LUMO of DMAD is also small (2.4 kcal mol^−1^) facilitating intermolecular charge transfer thereby resulting in the formation of the CT complex discussed later.

### Intramolecular charge transfer in CCMB 14

As mentioned earlier, in CCMB 14, positive and negative charges are exclusively restricted to the pyridinium and pyrrole rings respectively, and these two parts are intervened by the pyrazine ring. Intramolecular charge transfer (ICT) is an important phenomenon that exists in the molecules such as *p*-dimethylaminobenzonitrile which have both electron-donating (D) and electron-accepting (A) substituent groups.^32^ Apart from many other characteristics associated with such molecules, ICT was found to be accompanied by twisting of conformation termed as twisted intramolecular charge transfer abbreviated as TICT.^33^ In the present case also, the central ring is found to be folded like a boat making the positively and negatively charged fragments of the molecule facing each other.

### Time-dependent density functional theory (TDDFT) calculations

During the last two decades, TDDFT calculations have turned out to be increasingly popular for studying properties of the electronically excited states (EES).^34^ Besides studying various properties of the EES, amount of charge transfer and change in geometric parameters as a result of photon absorption by the CT complexes could be determined successfully.^35,36^

We carried out TDDFT calculations of CCMB 14 at the wB97XD/6-311+G(d,p) level.

**Fig. 5:**
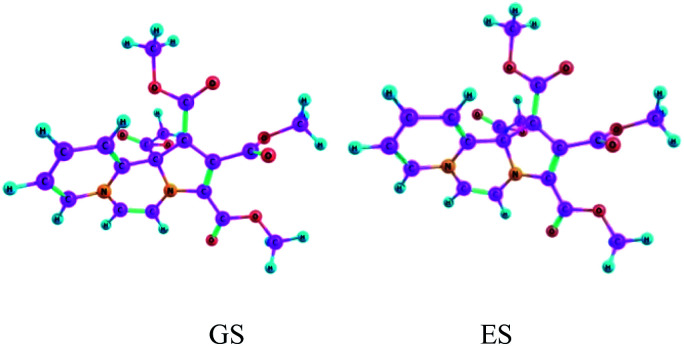
The ground state (GS) and the excited state (ES) geometries of CCMB 14 computed at the wB97XD/6-311+G(d,p) level.

Vertical excitation energies are found to be shifted slightly bathochromically as compared to the experimental values, but overall they are in reasonably good agreement. Equilibrium structures obtained for the electronic ground state and the first excited state of CCMB 14 are shown in Fig. 5.

ICT is accompanied by structural distortions.† It was found that on excitation, there are appreciable changes in the geometric parameters.

Molecular electrostatic potential (MEP) surface diagram is a useful tool to predict qualitatively electrophilic and nucleophilic sites in a molecule.^37,38^ It depicts electron density with varying colours, the regions of the highest and the least electron densities being shown in red and blue colours respectively. The electron densities decrease in the order: red > orange > yellow > green > blue.

**Fig. 6:**
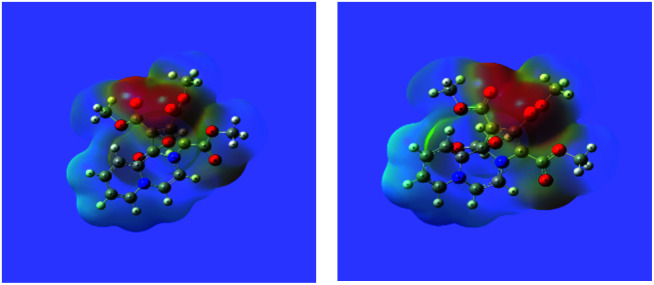
The MEP maps of the 14 in the ground and excited states calculated at the wB97XD/6-311+G(d,p) level.

The MEP maps of 14 in the ground (GS) and the excited (ES) states as determined by the TDDFT calculations are shown in Fig. 6.

It may be noted that density of the negative charge (intensity of the red colour) is much greater on the five-membered ring, which further increases in the excited state showing intramolecular charge transfer.

### Intermolecular charge transfer from CCMB 14 to DMAD

**Fig. 7:**
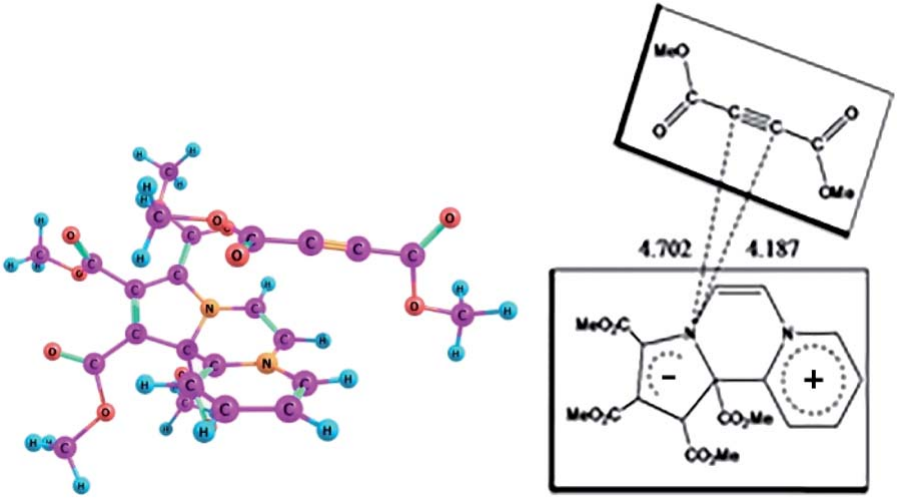
Optimized geometry of the CT complex between CCMB and DMAD in the ground state at the wB97XD/6-311+G(d,p) level.

As mentioned earlier, in the ^1^H NMR spectrum of the product, extra NMR signals are observed in the region – *δ* 3.8 ppm possibly due to additional DMAD molecules entrapped by 14 as CT complex. We optimized the ground state geometry of the 14-DMAD CT complex, which is shown in Fig. 7.

Formation of the CT complex is accompanied by lowering of the enthalpy, Δ*H*^0^ = −12.85 kcal mol^−1^ (Δ*G*^0^ = −0.43 kcal mol^−1^) calculated in the gas phase at the wB97XD/6-311+G(d,p) level.

The summary of the results of the TDDFT calculations of the CT complex, whose ground state (GS) geometry is shown in Fig. 7, are given in Table 1.

**Table 1:** Excitation energies and transition strengths of five low-lying excited states of 14

Excited states	Excitation energies (eV)	Excitation energies (nm)	Oscillator strength (*f*)
1	2.209	561.27	0.126
2	3.212	386.04	0.044
3	3.495	354.71	0.011
4	3.552	349.08	0.197
5	3.813	325.13	0.001

**Fig. 8:**
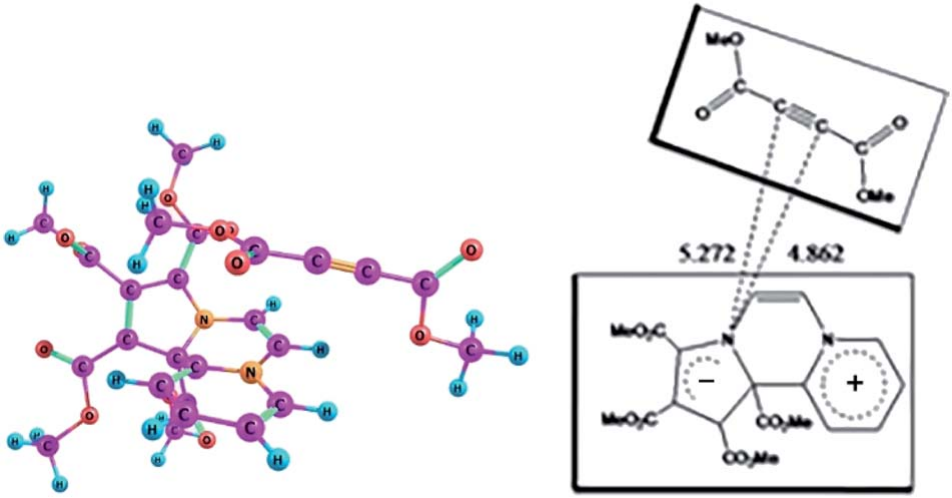
Optimized geometry of the CT complex between CCMB and DMAD in the excited state at the wB97XD/6-311+G(d,p) level.

On the basis of the oscillator strengths, two excited states, namely 1 and 4 are noteworthy. The *λ*_max_ values of these excited states at 561 nm and 349 nm respectively are, however, somewhat greater than the experimentally observed values of 518 nm and 346 nm respectively. It has been reported earlier that TDDFT calculations underestimate CT excitation energies.^39^ Optimized geometry of the CT complex in the excited state is shown in Fig. 8.

**Fig. 9:**
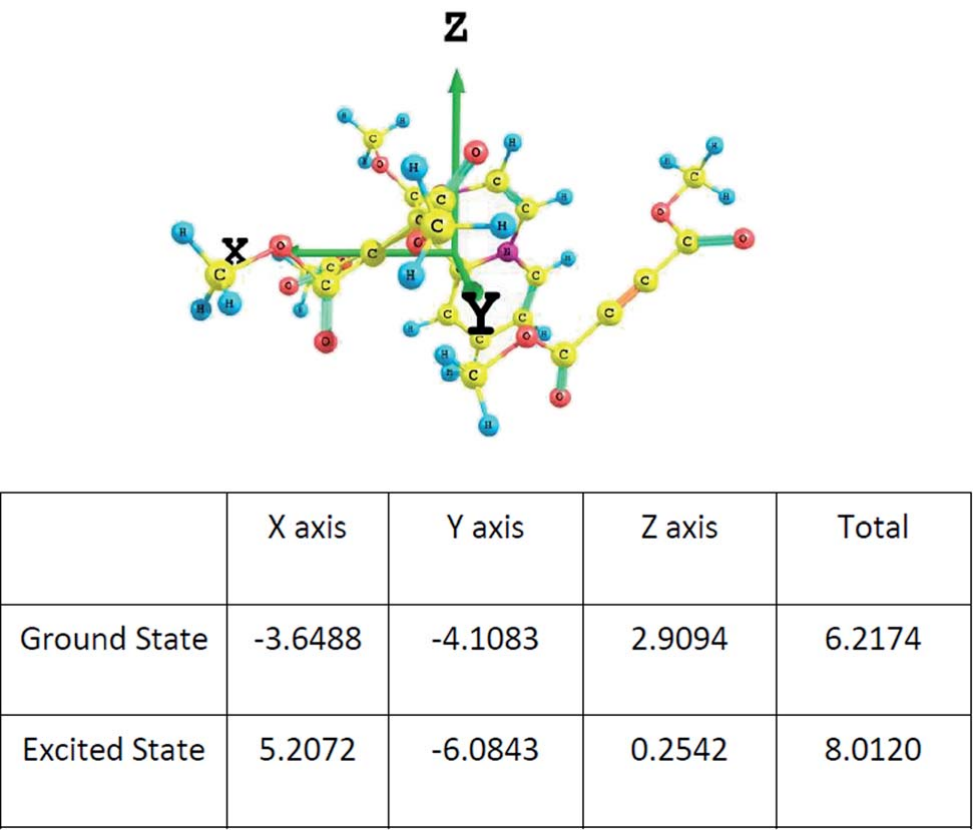
The components of the dipole moment of the CT complex along three axes determined at the wB97XD/6-311+G(d,p) level.

The dipole moments of the CT complex in the ground and excited states along three axes are given in Fig. 9.

It may be noted that there is substantial increase in the total value of the dipole moment in the excited state indicating charge transfer along the same axis as in the ground state.

### Computation of the model reaction sequence

**Fig. 10:**
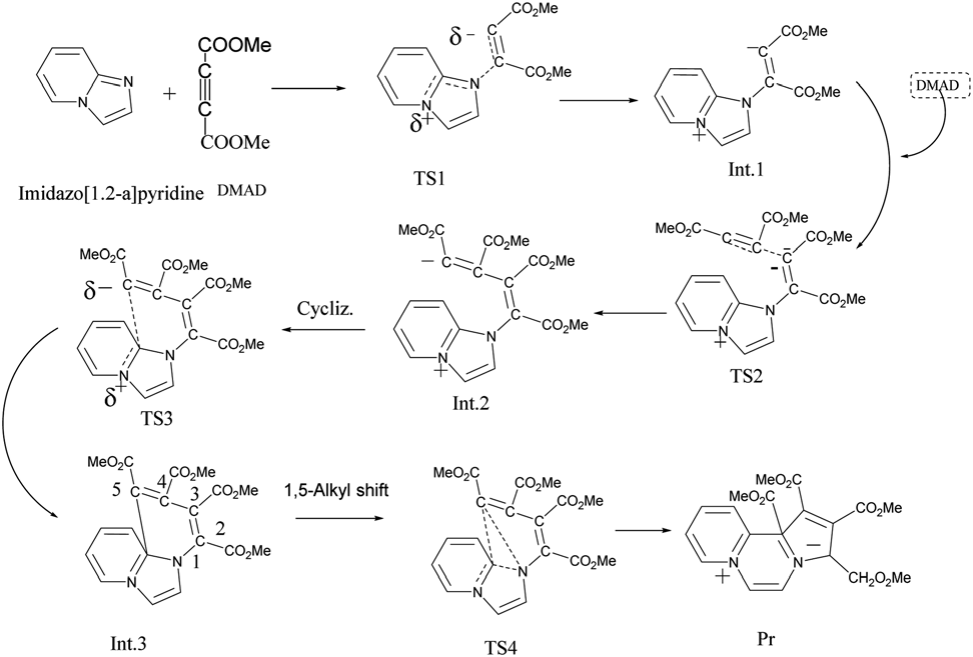
Model reaction sequence computed at the B3LYP/6-31+G(d) level.

As mentioned in the beginning, the reaction of imidazo[1,2-*a*]pyridine with DMAD is highly exothermic and very fast. For understanding and rationalising these features, following sequence of the model reactions was computed (Fig. 10).

**Fig. 11:**
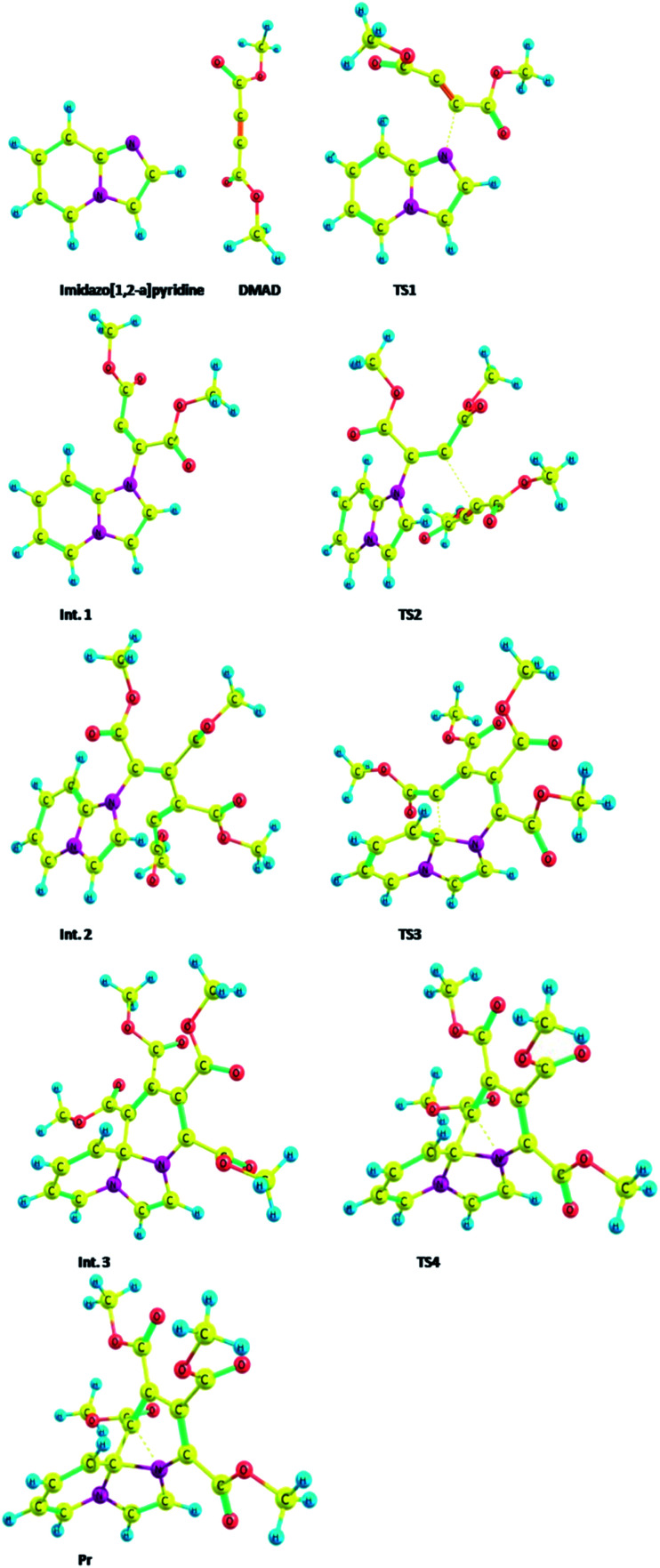
Optimized geometries of the species participating in the reactions sequence shown in Scheme 4 computed at the B3LYP/6-31+G(d) level.

Optimized geometries of all the species are shown in Fig. 11.

Thermodynamic data of the above sequence of reactions are given in the ESI Table S1.†

**Fig. 12:**
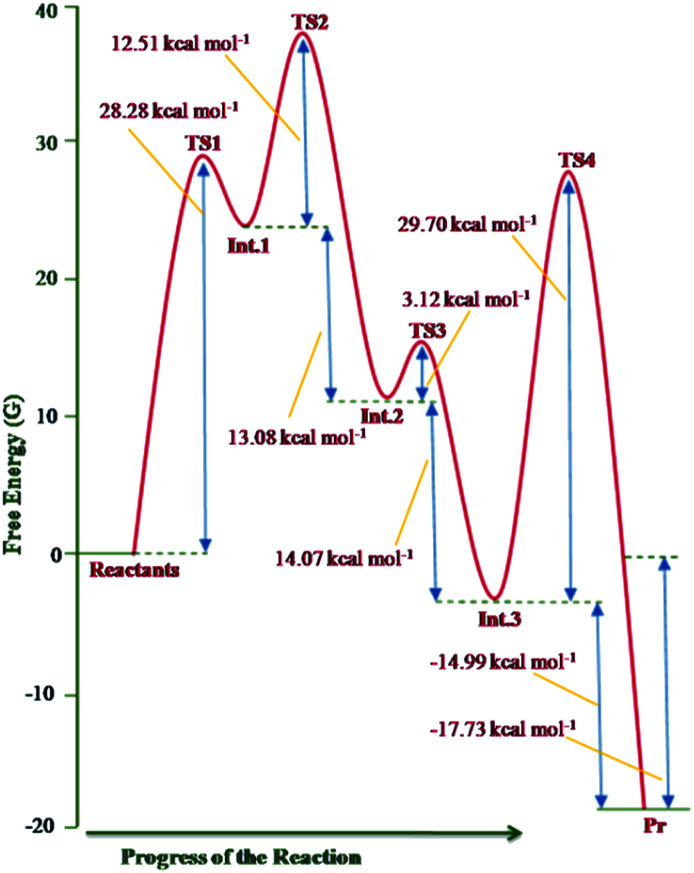
Free energy profile of the reaction sequence between imidazo[1,2-*a*]pyridine and DMAD computed at the B3LYP/6-31+G(d) level.

The free energy profile of the reaction is shown in Fig. 12.

The theoretical results rationalise the experimental observations and results. From Fig. 12, it may be inferred that formation of the transition structure TS2 would be the rate-limiting step with Gibbs free energy activation barrier (Δ*G*^#^) of 36.9 kcal mol^−1^. This is rather a high energy barrier, which possibly results due to overestimated translation entropy in TS2 in the gas phase wherein three independent fragments are arranged. As one of the reviewers opined, this problem is often faced while calculating Gibbs free energies of species having different number of atoms. In view of this, the possibility of the TS1 as the rate-limiting step cannot be ruled out completely. It is noteworthy that overall reaction is highly exothermic (Δ*H*^0^ = −49.2 kcal mol^−1^) and exergonic (Δ*G*^0^ = −17.7 kcal mol^−1^), the facts in consonance with the experimental observation. The driving force for the conversion of the initially formed covalent species 13 (Int. 3) into CCMB 14 (Pr) is the release of much energy (Δ*H*^0^ = −15.5 kcal mol^−1^, Δ*G*^0^ = −15.0 kcal mol^−1^). The greater thermodynamic stability of 14 can be attributed to the extensive delocalisation of the positive and negative charges in the respective parts of the molecule.

## Conclusions

A cascade reaction sets in on adding DMAD into imidazo[1,2-*a*]pyridine solution to give a new CCMB. The ^1^H NMR spectrum reveals presence of traces of DMAD possibly bound in the form of the CT complex with the CCMB. Formation of the CT complex could be established spectrophotometrically. The TDDFT calculations reveal existence of both intramolecular as well as intermolecular charge transfer. The electronic excitations of the product and the CT complex could be rationalised by the TDDFT calculations. A theoretical investigation of the reaction at the DFT level reveals that it occurs in four steps.

It provides a facile method for the synthesis of a new class of the CCMBs isoconjugate with odd non-alternant hydrocarbon anion.

## Experimental section

### General details

Commercially available imidazo[1,2-*a*]pyridine and DMAD were directly used without further purification. Solvents were freshly dried and distilled according to the known procedure. Melting point was measured in open capillary and is uncorrected. The UV-visible spectra were recorded on Shimadzu 160 UV-vis spectrophotometer in the range of 200–800 nm with a quartz cell of 1 cm path length. The IR spectrum of the compound was recorded on Bruker FT IR spectrometer ALPHA II in KBr pellet. The wave numbers of the recorded IR signals are quoted in cm^−1^. The ^1^H NMR spectrum was obtained at 25 °C on Jeol Resonance JNM-ECS400 DELTA2_NMR-400 MHz spectrometer and ^13^C NMR and COSY spectra were scanned on Bruker-DPX-300 MHZ spectrometer in CDCl_3_ with TMS as an internal reference. All the chemical shifts are reported in parts per million (*δ* ppm). Coupling constants (*J*) are given in Hertz. Proton spectral multiplicities are abbreviated as – s: singlet, d: doublet, t: triplet, m: multiplet, q: quartet. Low resolution mass spectrum was recorded on Aligent G1946 LC-MS. For this, the sample was dissolved in methanol and after filtering through 0.45 micron nylon filter, it was inserted directly into the ESI ion source. High resolution mass spectrum (HRMS) was recorded with a Waters Xevo G2-S QTOF instrument by directly injecting the sample dissolved in 2 mL methanol.

### Reaction of DMAD with imidazo[1,2-*a*]pyridine: synthesis of tetramethyl pyrrolo[1,2-*a*][2′,1′-*c*]pyridopyrazinium-1,2,3,4-tetracarboxylate

Oven dried glassware was used and the experiment was carried out under nitrogen atmosphere. To imidazo[1,2-*a*]pyridine [96.1 mg, 0.81 mmol, 80 μL] dissolved in diethyl ether [5 mL] and placed in a 25 mL round bottom (RB) flask, a solution of DMAD [231 mg, 1.62 mmol, 0.2 mL] in diethyl ether [5 mL] was added drop-wise under stirring at 10–15 °C by using a dropping funnel. After addition of a few drops, there was immediate appearance of pink colour. The drop-wise addition was kept very slow and completed in 30 minutes. After completion, the stirring was continued for 1 h while maintaining the temperature at 10–15 °C. The deposited brown solid was separated by filtration under nitrogen in a sintered funnel. From here onwards, the compound was transferred to a side tube 50 mL RB flask and was macerated with diethyl ether by maintaining nitrogen cushion in flask and the solid was washed with diethyl ether (3 × 5 mL) and dried *in vacuo*. Yield: 0.25 g, 78.5%. Mp 124–126 °C. IR (KBr): *ν*(cm^−1^) 2919, 2851, 1734, 1464, 1374, 1265, 1094 cm^−1^. ^1^H NMR (400 MHz, CDCl_3_) *δ* (ppm) 8.11 (d, ^3^*J*_HH_ = 6.4 Hz), 7.61 (d, ^3^*J*_HH_ = 6.8 Hz), 7.55 (t, ^3^*J*_HH_ = 6.8 Hz), 7.15 (d, ^3^*J*_HH_ = 5.6 Hz), 7.13 (d, ^3^*J*_HH_ = 5.6 Hz), 6.77 (t, ^3^*J*_HH_ = 6.8 Hz), 3.71 (s, 3H), 3.70 (s, 3H), 3.66 (s, 3H), 3.65 (s, 3H). ^13^C NMR (75 MHz, CDCl_3_) *δ* (ppm) = 165.37, 165.15, 162.55, 138.56, 136.76, 136.39, 132.50, 126.10, 112.73, 58.91, 54.29, 53.90, 53.50, 53.01, 52.88, 52.64, 52.54, 52.51, 52.12, 51.95, 51.90, 50.96. HRMS (ESI-TOF) *m*/*z*: calcd for C_19_H_19_N_2_O_8_: 403.114 [M + H]^+^; found 403.116.

### Preparation of the stock solutions

#### Acceptor (stock solution 10^−2^ M)

By dissolving DMAD (14.21 mg, 0.1 mmol) in dichloromethane taken in 10 mL volumetric flask and made up to the mark.

#### Donor (stock solution 10^−2^ M)

By dissolving imidazo[1,2-*a*]pyridine (11.8 mg, 0.1 mmol, 12 μL) in dichloromethane taken in 10 mL volumetric flask and made up to the mark.

**Table 2:** Molar compositions of the solutions used for UV-vis spectroscopy

Solution no.	I	II	III	IV	V	VI	VII	VIII
Imidazo[1,2-*a*]pyridine solution (mL)	1.0 mL (0.01 mmol)	1.0 (0.01 mmol)	1.0 (0.01 mmol)	1.0 (0.01 mmol)	1.0 (0.01 mmol)	1.0 (0.01 mmol)	1.0 (0.01 mmol)	1.0 (0.01 mmol)
DMAD solution (mL)	0	0.5 (0.005 mmol)	1.0 (0.01 mmol)	1.5 (0.015 mmol)	2.0 (0.02 mmol)	2.5 (0.025 mmol)	3.0 (0.03 mmol)	3.5 (0.035 mmol)
Dichloromethane (mL)	9.0	8.5	8.0	7.5	7.0	6.5	6.0	5.5

## Conflicts of interest

There are no conflicts to declare.

## Supplementary Material

RA-011-D1RA03981D-s001
