# Supplementary material for: A new cross-conjugated mesomeric betaine
Source: RSC Adv. 2021 Jul 21;11(41):25296–304. doi: 10.1039/d1ra03981d (PMC9037020; doi:10.1039/d1ra03981d)
Supplement: RA-011-D1RA03981D-s001 [file RA-011-D1RA03981D-s001.pdf]

Electronic Supplementary Material (ESI) for RSC Advances.
This journal is © The Royal Society of Chemistry 2021

Supporting Information

for

A new cross-conjugated mesomeric betaine

Nivedita Sharma, Manjinder Kour, Raakhi Gupta, Raj K. Bansal*

Department of Chemistry, The IIS (deemed to be University), Jaipur 302020, India

Table of Contents **Page**

General Information S2

Reaction of DMAD with Imidazo[1,2-*a*]pyridine S2

Computational Details and References S3-S4

IR Spectrum S4

^{1}H NMR Spectra S4-S5

2D COSY NMR Spectrum S6

^{13}C NMR Spectrum S7

Low Resolution Mass Spectrum S8

High Resolution Mass Spectrum S9

Thermodynamic data of different species involved in the reaction of imidazo[1,2-*a*]pyridine with DMAD Table DMAD Table S1 S9

Total Energies and Cartesian Coordinates of the Optimized Structures Table S2 S10-26

Experimental Section

General Information

Commercially available imidazo[1,2-*a*]pyridine and DMAD were directly used for the synthesis without further purification. Solvents were freshly dried and distilled according to the known procedure. Melting point was measured in open capillary and is uncorrected. The UV-visible spectra were recorded on Shimadzu 160 UV-vis spectrophotometer in the range of 200-800 nm with a quartz cell of 0.1 cm path length. The infrared spectrum of the compound was recorded on Bruker FT IR spectrometer ALPHA II in KBr pellet. The wave numbers of recorded IR signals are quoted in cm^{-1}. ^{1}H NMR spectrum were obtained at 25˚C using Jeol Resonance JNM-ECS400 DELTA2_NMR-400MHz spectrometer and ^{13}C NMR and COSY using Bruker-DPX-300 MHZ spectrometer in deuterated solvent ($CDCl_3$) with TMS as an internal reference. All the chemical shifts are reported in parts per million (δ ppm). Coupling constants (*J*) are given in Hertz. Proton spectral multiplicities are abbreviated as follows; s: singlet, d: doublet, t: triplet, m: multiplet, q: quartet. Low resolution mass spectrum was recorded on Aligent G1946 LC-MS. For this, the sample was dissolved in methanol and after filtering through 0.45 micron nylon filter, it was inserted directly into the ESI ion source. High resolution mass spectrum (HRMS) was recorded with a Waters Xevo G2-S QTOF instrument by directly injecting the sample dissolved in 2 mL methanol.

Reaction of DMAD with Imidazo[1,2-*a*]pyridine

Oven dried glassware was used and the experiment was carried out under nitrogen atmosphere. To imidazo [1,2-*a*]pyridine [96.1 mg, 0.81 mmol, 0.08 mL] dissolved in diethyl ether [5 mL] and placed in a 25 mL round bottom (RB) flask, a solution of DMAD [231 mg, 1.62 mmol, 0.2 mL] in diethyl ether [5 mL] was added drop-wise under stirring at 10-15 °C by using a dropping funnel. After addition of a few drops, there was immediate appearance of pink colour. The drop-wise addition was kept very slow and completed in 30 minutes. After completion, the stirring was continued for 1 hr while maintaining the temperature at 10-15°C. A brown solid was separated by filtration under nitrogen in a sintered funnel. From here onwards, the compound was transferred to a side tube round bottom (RB) flask. The compound was macerated by maintaining nitrogen cushion in flask and the solid was washed with diethyl ether (1×5 mL) and dried in vacuo. Yield: 0.25 g, 78.5%. m.p. 124-126˚C. IR (KBr): 2919, 2851, 1734, 1464, 1374, 1265, 1094 cm^{-1}. ^{1}H NMR ($CDCl_3$, 400 MHz) δ (ppm) = 8.17 (d, $^{3}J_{HH}$= 6.4 Hz), 7.61 (d, $^{3}J_{HH}$= 6.8 Hz), 7.55 (t, $^{3}J_{HH}$ = 6.8 Hz), 7.15 (d, $^{3}J_{HH}$ = 5.6 Hz), 7.13 (d, $^{3}J_{HH}$ = 5.6 Hz), 6.77 (t, $^{3}J_{HH}$ = 6.8 Hz), 3.71 (s, 3H), 3.70 (s, 3H), 3.66 (s, 3H), 3.65 (s, 3H) ppm. ^{13}C NMR ($CDCl_3$, 75 MHz) δ (ppm) = 165.37, 165.15, 162.55, 138.56, 136.76, 136.39, 132.50, 126.10, 112.73, 58.91, 54.29, 53.90, 53.50, 53.01, 52.88, 52.64, 52.54, 52.51, 52.12, 51.95, 51.90, 50.96. HRMS (ESI-TOF) m/z: $[M+H]^+$ Calcd for $C_{19}H_{19}N_2O_8$ 403.114; found 403.116.

Computational Details

Gaussian16 suite of program was used for all calculations [29]. We computed the model reactions sequence using density functional calculations without dispersion correction (B3LYP). Here, split valence basis sets with polarized and diffused functions on heavy atoms, 6-31+G(d) were used. Thus, geometries of the reactants, products and transition structures involved in reaction were optimized in the gas phase at the B3LYP/6-31+G(d) level. Frequency calculations were done at the same level to characterize energy minimum or the first saddle point by presence of one or no imaginary frequency respectively. The intrinsic reaction coordinate (IRC) [30,31] calculations starting from the transition structure were carried out at the same theory level to confirm its (transition structure) connection to the respective reactant and the intermediate/product. Total enthalpy was calculated by adding thermal correction to the sum of the electronic and thermal enthalpy. The free energy was calculated at temperature 298.15 K as follows:

$\Delta G = \Delta H - T\Delta S$

ΔH = relative enthalpy

ΔS = relative entropy

T = 298.15 K

Natural Bond Orbital (NBO) [32] calculations were done at the same level of theory. Computational calculations for the charge transfer complex both in the ground state and the excited state (TDDFT calculations) were done at the wB97XD/6-311+G(d,p) level [33].

References

[29] Gaussian 16, Revision C.01, Frisch, M. J.; Trucks, G. W.; Schlegel, H. B.; Scuseria, G. E.; Robb, M. A.; Cheeseman, J. R.; Scalmani, G.; Barone, V.; Petersson, G. A.; Nakatsuji, H.; Li, X.; Caricato, M.; Marenich, A. V.; Bloino, J.; Janesko, B. G.; Gomperts, R.; Mennucci, B.; Hratchian, H. P.; Ortiz, J. V.; Izmaylov, A. F.; Sonnenberg, J. L.; Williams-Young, D.; Ding, F.; Lipparini, F.; Egidi, F.; Goings, J.; Peng, B.; Petrone, A.; Henderson, T.; Ranasinghe, D.; Zakrzewski, V. G.; Gao, J.; Rega, N.; Zheng, G.; Liang, W.; Hada, M.; Ehara, M.; Toyota, K.; Fukuda, R.; Hasegawa, J.; Ishida, M.; Nakajima, T.; Honda, Y.; Kitao, O.; Nakai, H.; Vreven, T.; Throssell, K.; Montgomery, J. A.; Jr., Peralta, J. E.; Ogliaro, F.; Bearpark, M. J.; Heyd, J. J.; Brothers, E. N.; Kudin, K. N.; Staroverov, V. N.; Keith, T. A.; Kobayashi, R.; Normand, J.; Raghavachari, K.; Rendell, A. P.; Burant, J. C.; Iyengar, S. S.; Tomasi, J.; Cossi, M.; Millam, J. M.; Klene, M.; Adamo, C.; Cammi, R.; Ochterski, J. W.; Martin, R. L.; Morokuma, K.; Farkas, O.; Foresman, J. B.; and Fox, D. J. Gaussian, Inc., Wallingford CT, **2016**.
[30] Gonzalez, C.; Schlegel, H. B. *J. Chem. Phys.* **1989**, *90*, 2154-2161 doi:10.1063/1.456010
[31] Gonzalez, C.; Schlegel, H. B. *J. Phys. Chem.* **1990**, *94*, 5523-5527. doi:10.1021/j100377a021

[32] Glendening, E. D.; Reed, A. E.; Carpenter, J. E. Weinhold, F. *An improved, F. NBO version 3.1*. **2001**.

[33] Chai, J.; Head-Gordon, M. *Phys. Chem. Chem. Phys*. **2008**, *10*, 6615-6620. doi:10.1039/B810189B

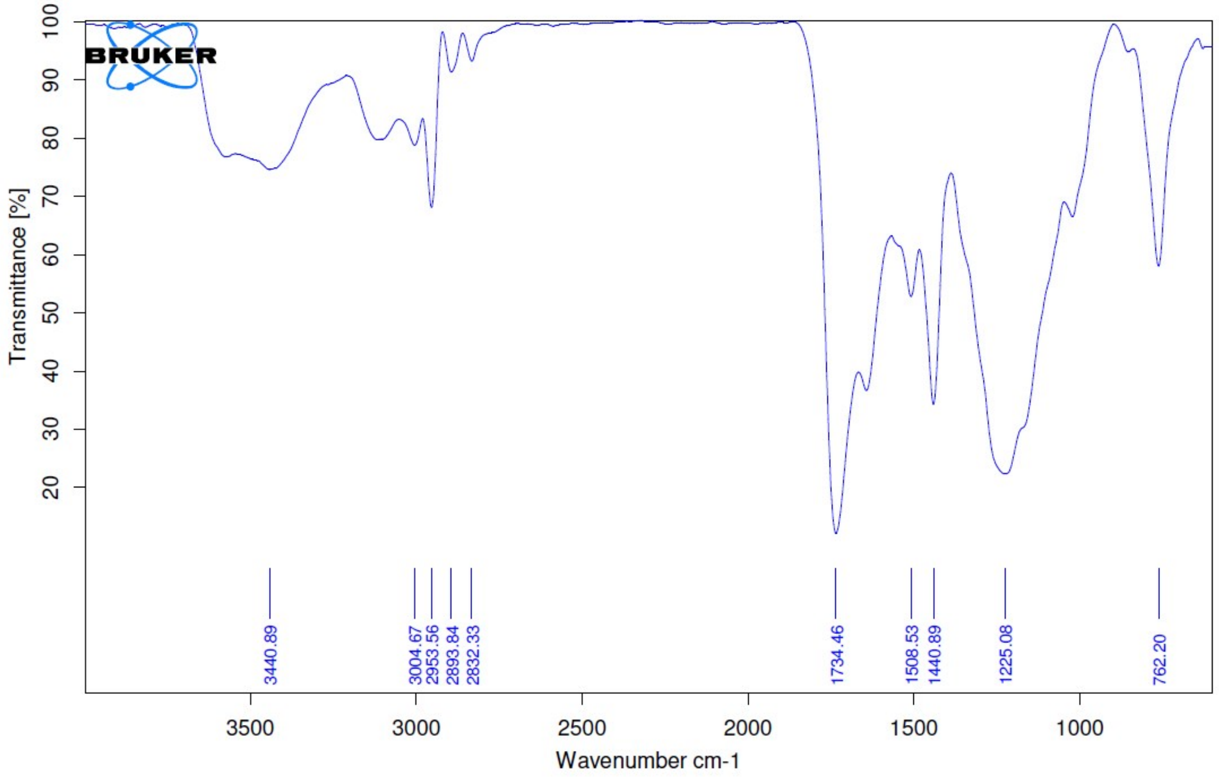

Figure S1. IR spectrum recorded on Bruker FT IR spectrometer.

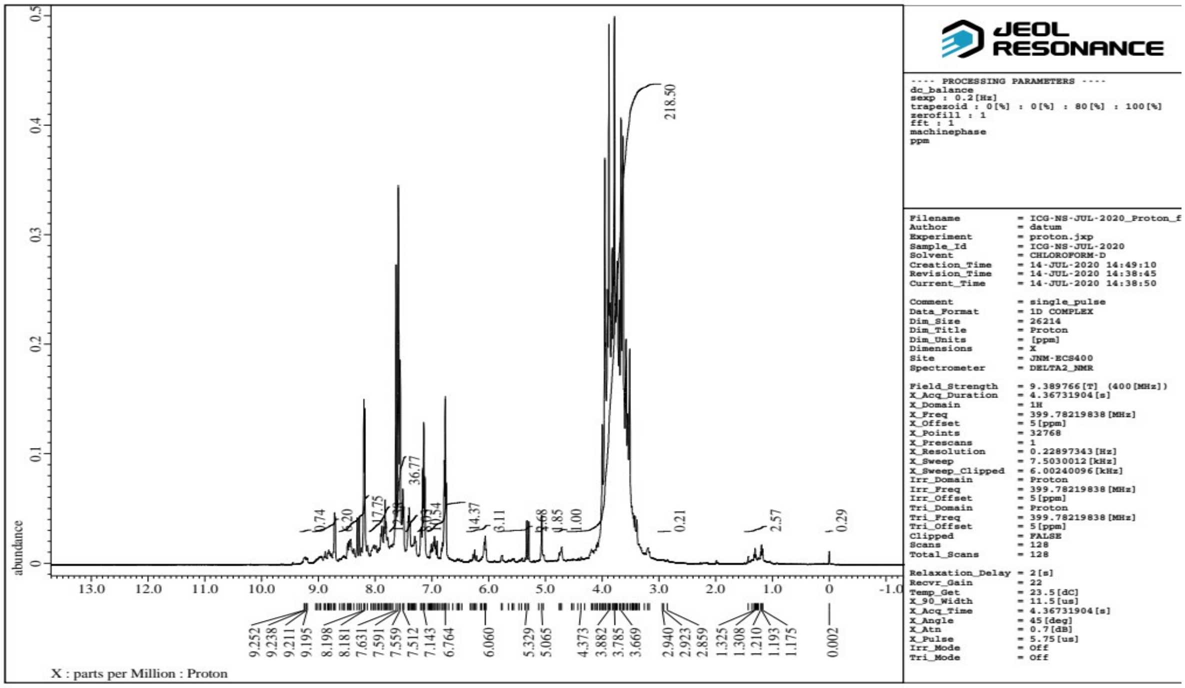

Figure S2. ^{1}H NMR spectrum taken at 25 °C in $CDCl_3$ at 300 MHz.

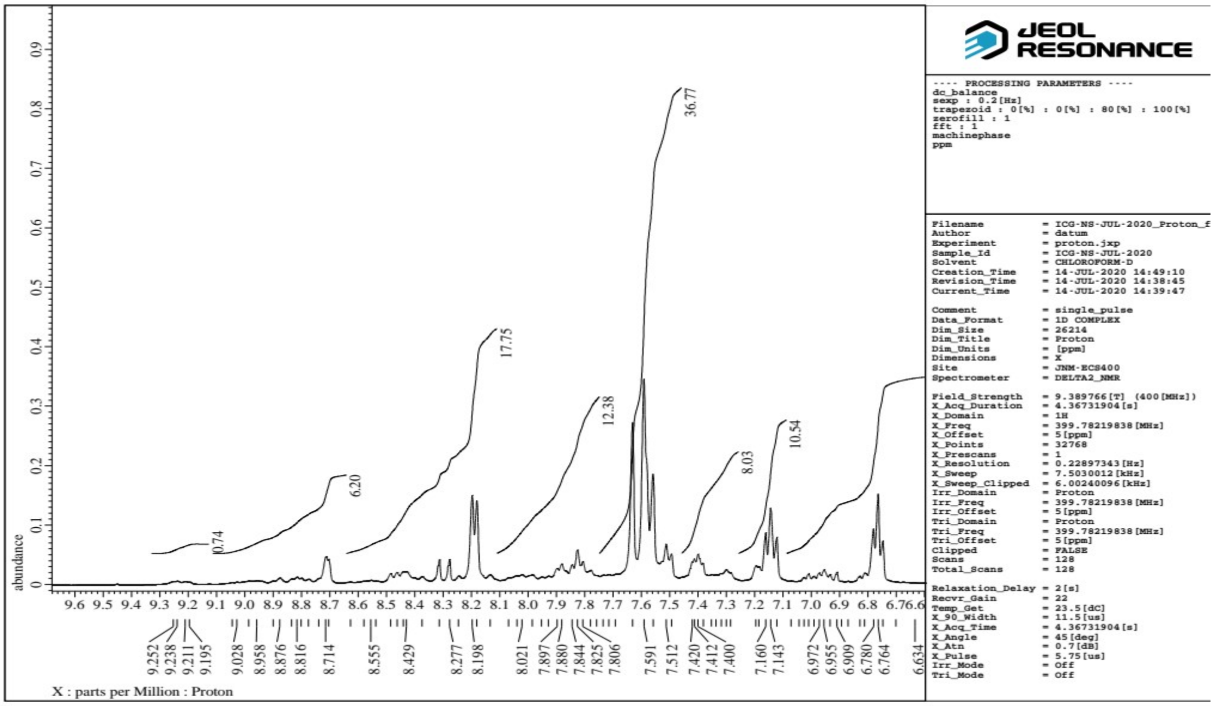

Figure S3. Expended ^{1}H NMR spectrum (6.6-9.6 ppm) taken at 25 °C in CDCl3 at 300 MHz.

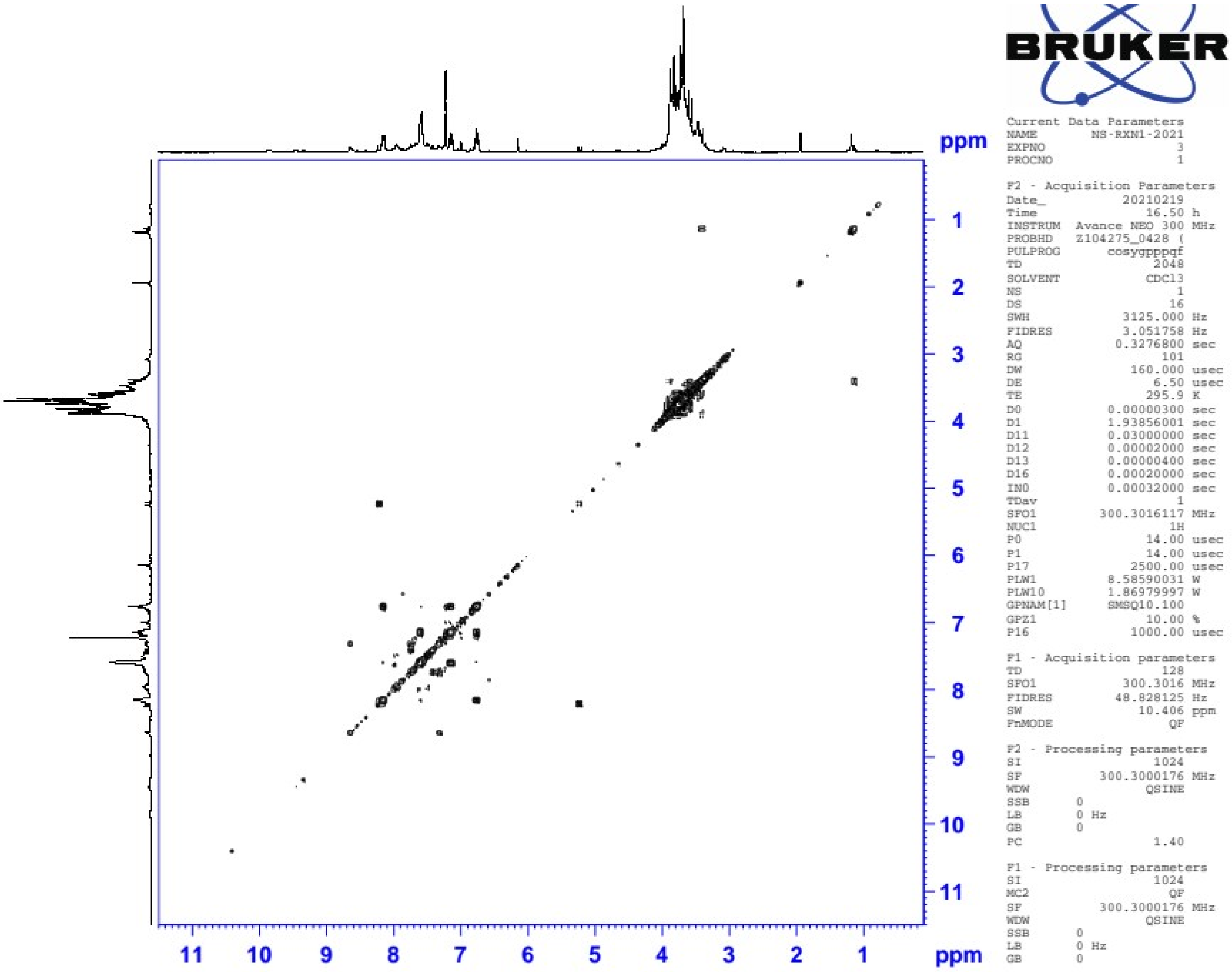

Figure S5. ^1H-^1H Correlation spectrum (2D-COSY) taken at 25 °C in CDCl3 at 300 MHz.

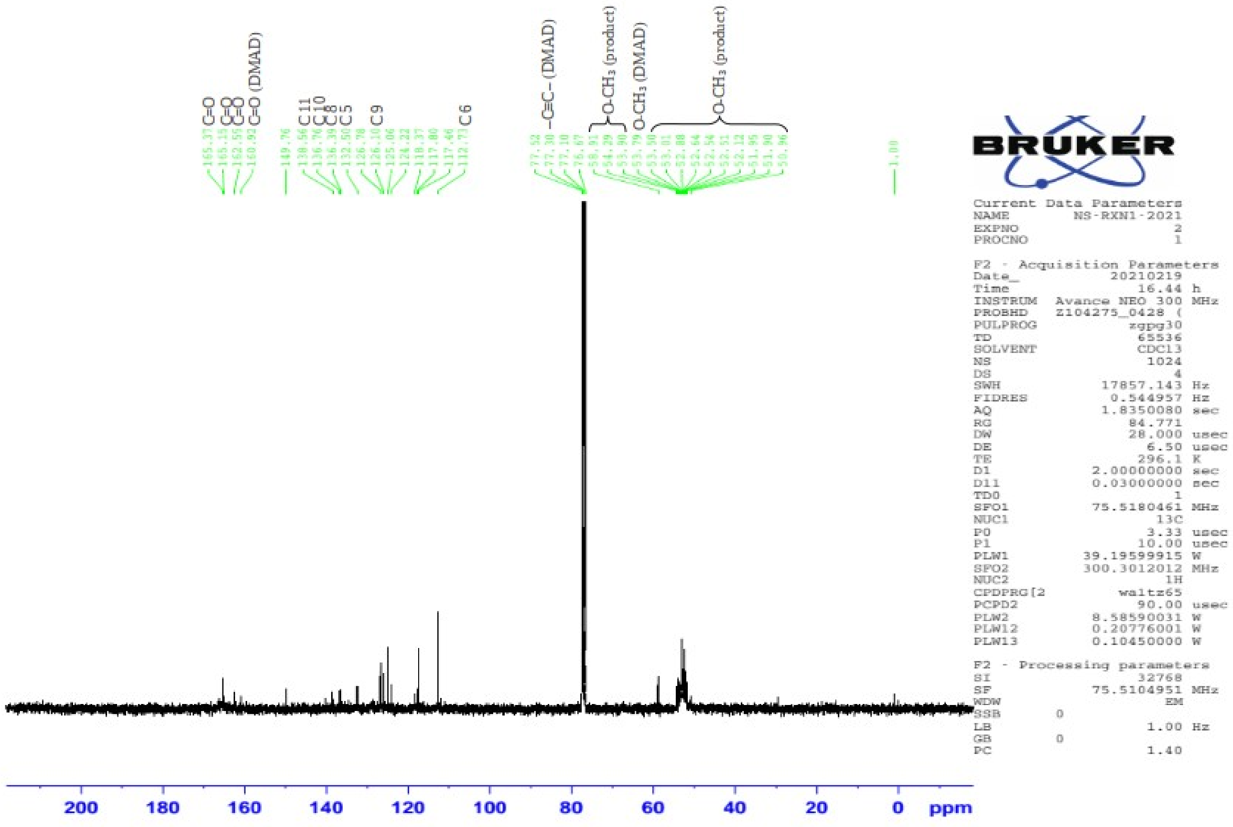

Figure S6. ^{13}C NMR spectrum taken at 25 °C in CDCl3 at 300 MHz.

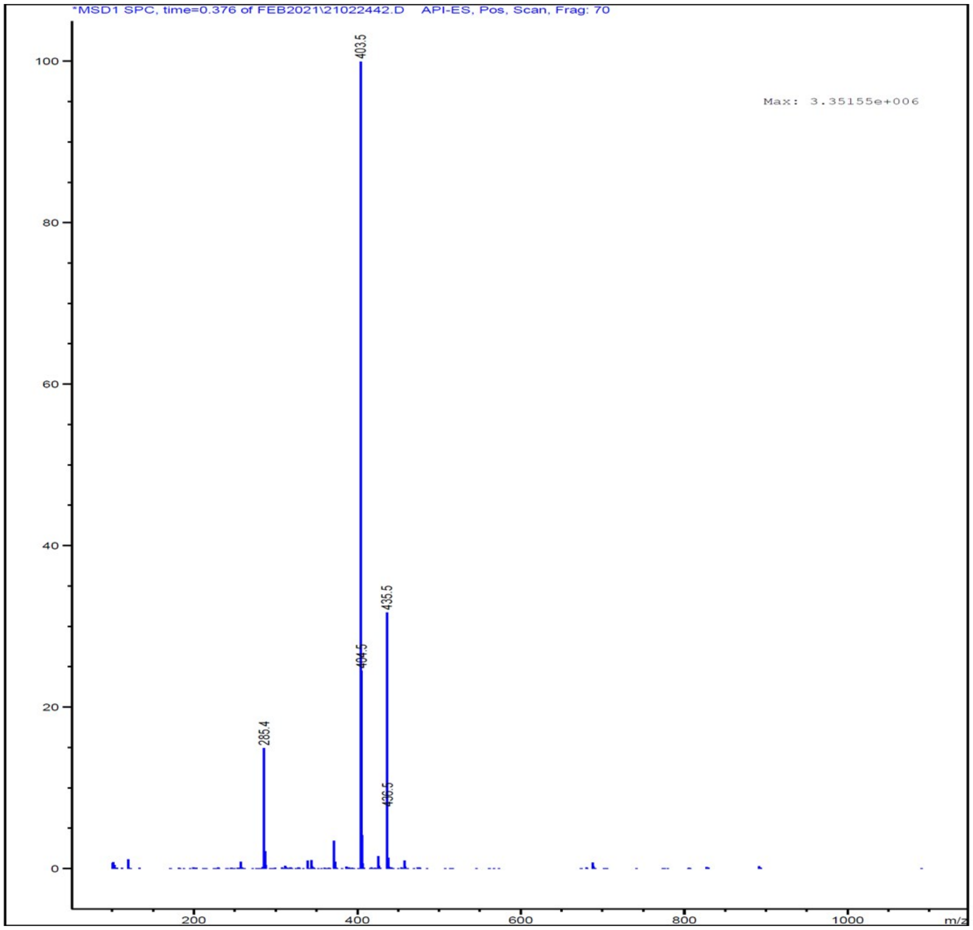

Figure S7. Low resolution mass spectrum recorded on Aligent G1946 LC-MS.

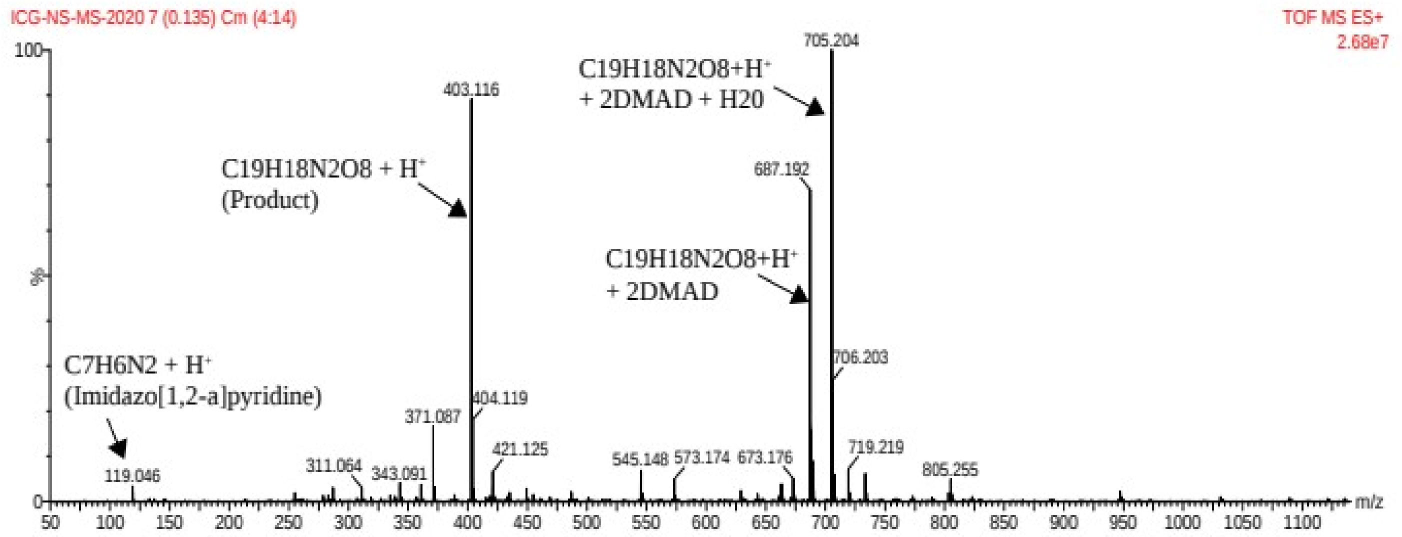

Figure S8. HRMS recorded with a Waters Xevo G2-S QTOF instrument.

Table S1. Thermodynamic data of different species involved in the reaction of imidazo[1,2-*a*]pyridine with DMAD calculated in the gas phase at the B3LYP/6-31+G(d) level.

Species	Total Enthalpy H (a.u.)	Entropy S (Cal K^{-1} mol^{-1})	Activation Enthalpy $\Delta H^{\#}$ (kcal mol^{-1})	Activation Entropy $\Delta S^{\#}$ (kcal $K^{-1}mol^{-1}$)	Activation Free Energy $\Delta G^{\#}$ (kcal mol^{-1})	Standard Enthalpy ΔH^0 (kcal mol^{-1})	Standard Free Energy ΔG^0 (kcal mol^{-1})
DMAD	-532.845939	106.502	-	-	-	-	-
Imidazo[1,2-*a*]pyridine	-379.615021	77.986	-	-	-	-	-
TS1	-912.436643	140.802	15.3	-0.043	28.3	-	-
Int1	-912.444013	138.242	-	-	-	10.6	24.41
TS2	-1445.2917	199.128	-1.1	-0.045	12.51	-	-
Int2	-1445.333403	197.171	-	-	-	-27.3	-13.1
TS3	-1445.331514	190.704	1.2	-0.006	3.11	-	-
Int3	-1445.360482	187.392	-	-	-	-17.0	-14.1
TS4	-1445.313702	186.264	29.35	-0.001	29.7	-	-
Pr	-1445.385236	185.576	-	-	-	-49.2	-17.7

Table S2. Total energies, number of imaginary frequencies and Cartesian coordinates of the optimized geometries of different species

DMAD

Total energy -532.984949 a.u.

Number of imaginary frequencies 0

6	0.602950000	-0.199994000	-0.058257000
6	-0.602948000	-0.199999000	0.058237000
6	2.034769000	-0.250623000	-0.278752000
6	-2.034769000	-0.250696000	0.278700000
8	2.682740000	0.602731000	0.531861000
8	2.560844000	-0.977136000	-1.096670000
6	4.120267000	0.628038000	0.390874000
1	4.461739000	1.366934000	1.115153000
1	4.537156000	-0.357515000	0.612486000
1	4.393564000	0.921895000	-0.625567000
8	-2.682710000	0.603092000	-0.531480000
8	-2.560893000	-0.977807000	1.096055000
6	-4.120251000	0.628243000	-0.390611000
1	-4.461695000	1.367539000	-1.114494000
1	-4.537069000	-0.357203000	-0.612830000
1	-4.393656000	0.921498000	0.625975000

Imidazo[1,2-a]pyridine

Total energy -379.746741 a.u.

Number of imaginary frequencies 0

7	-1.899045000	-0.490793000	0.000000000

6	-0.567828000	-0.554973000	0.000000000
6	0.303979000	-1.669821000	0.000000000
6	1.664477000	-1.468481000	0.000000000
6	2.196743000	-0.144950000	0.000000000
6	1.360036000	0.937005000	0.000000000
7	0.000000000	0.736339000	0.000000000
1	-0.137507000	-2.660691000	0.000000000
1	2.342639000	-2.316507000	0.000000000
1	3.269110000	0.019252000	0.000000000
1	1.697179000	1.967448000	0.000000000
6	-1.055993000	1.625643000	0.000000000
6	-2.193559000	0.840713000	0.000000000
1	-3.219734000	1.185854000	0.000000000
1	-0.905506000	2.694999000	0.000000000

TS1

Total energy -912.707801 a.u.

Number of imaginary frequencies 1

6	-3.688977000	-1.379897000	0.128987000
7	-2.364877000	-1.473353000	0.487786000
6	-1.370520000	-0.728184000	-0.151529000
6	-1.732889000	0.160248000	-1.189017000
6	-3.059790000	0.252671000	-1.542081000
6	-4.047878000	-0.528386000	-0.878498000
7	-0.182342000	-1.038457000	0.391346000

6	-0.407377000	-1.964243000	1.371413000
6	-1.746752000	-2.250185000	1.455116000
6	1.397132000	-0.074207000	-0.006116000
6	2.389676000	-1.155608000	-0.124369000
8	2.222029000	-2.326043000	0.174249000
6	1.364667000	1.186813000	-0.073960000
6	0.649871000	2.408433000	-0.080706000
8	0.058161000	2.865449000	-1.058978000
8	3.556386000	-0.677038000	-0.601113000
6	4.617176000	-1.640168000	-0.723794000
8	0.720066000	3.064892000	1.112495000
6	0.077119000	4.348123000	1.151248000
1	-5.093033000	-0.454809000	-1.159032000
1	-3.357589000	0.935778000	-2.331206000
1	-0.971508000	0.772243000	-1.658758000
1	-4.380791000	-2.004237000	0.682084000
1	0.409685000	-2.383276000	1.937652000
1	-2.304004000	-2.922238000	2.089898000
1	5.463608000	-1.079592000	-1.121816000
1	4.862038000	-2.067560000	0.252718000
1	4.329233000	-2.443661000	-1.407457000
1	0.249288000	4.723431000	2.161246000
1	0.517047000	5.022992000	0.411287000
1	-0.995310000	4.253055000	0.956067000

Int. 1

Total energy -912.716893a.u.

Number of imaginary frequencies 0

6	-4.329993000	0.244241000	0.005773000
7	-3.146662000	-0.410599000	-0.242357000
6	-1.921549000	0.178812000	0.022528000
6	-1.860761000	1.478669000	0.561455000
6	-3.048128000	2.129537000	0.813003000
6	-4.295068000	1.507404000	0.527673000
7	-0.957625000	-0.708722000	-0.321632000
6	-1.566576000	-1.849054000	-0.794116000
6	-2.919745000	-1.682786000	-0.753362000
6	0.493411000	-0.422802000	-0.266013000
6	1.235580000	-1.575540000	0.305478000
8	0.732376000	-2.647172000	0.628941000
6	0.905422000	0.771303000	-0.697782000
6	2.263414000	1.226755000	-0.742216000
8	3.022607000	1.119663000	-1.700145000
8	2.548853000	-1.321491000	0.426510000
6	3.358383000	-2.395513000	0.928551000
8	2.599598000	1.951995000	0.380027000
6	3.894159000	2.563668000	0.348378000
1	-5.229212000	2.023812000	0.719523000
1	-3.033333000	3.131860000	1.228860000
1	-0.888816000	1.922649000	0.731123000

1	-5.235347000	-0.299868000	-0.234346000
1	-0.984908000	-2.697232000	-1.113637000
1	-3.728614000	-2.338790000	-1.033662000
1	4.376882000	-2.006397000	0.932425000
1	3.045504000	-2.672016000	1.939490000
1	3.283316000	-3.269352000	0.275193000
1	3.985348000	3.101115000	1.294861000
1	4.682583000	1.809155000	0.262971000
1	3.977850000	3.258183000	-0.493641000

TS2

Total energy **-1445.704659 a.u.**

Number of imaginary frequencies **1**

6	-0.185688000	2.355768000	0.222382000
6	-2.095712000	-0.934341000	-0.470392000
7	-0.830985000	-0.822855000	-0.944990000
6	0.392629000	-1.243392000	-0.263334000
6	0.418402000	-2.716036000	-0.034312000
8	1.520284000	-3.112389000	0.624410000
6	1.339285000	-0.333382000	-0.024802000
6	2.661678000	-0.555974000	0.510669000
6	0.792712000	1.875247000	-0.363655000
6	1.850118000	1.994824000	-1.366625000
8	3.012983000	2.379795000	-0.822727000
6	-1.386368000	2.481473000	0.965902000

7	-2.937796000	-0.368429000	-1.404574000
6	-2.611755000	-1.472845000	0.719454000
6	-3.972650000	-1.413600000	0.919123000
6	-4.821790000	-0.822915000	-0.053566000
6	-4.292077000	-0.302933000	-1.201962000
6	-2.169698000	0.108194000	-2.460903000
6	-0.872773000	-0.175645000	-2.161544000
8	-0.457416000	-3.484980000	-0.404203000
8	3.657107000	-0.747916000	-0.179944000
8	2.711809000	-0.384984000	1.859234000
6	4.024082000	-0.407006000	2.440624000
8	1.691462000	1.765620000	-2.555286000
8	-2.491144000	2.089371000	0.587992000
8	-1.199582000	3.110913000	2.156666000
6	-2.375918000	3.293274000	2.957552000
6	1.660676000	-4.528713000	0.827844000
6	4.149139000	2.400516000	-1.712552000
1	-5.892856000	-0.768137000	0.105348000
1	-4.401792000	-1.818733000	1.829949000
1	-1.938155000	-1.915588000	1.441484000
1	-4.867711000	0.178129000	-1.983001000
1	0.036227000	0.078357000	-2.687902000
1	-2.609635000	0.624016000	-3.299236000
1	2.614739000	-4.649664000	1.341129000
1	0.839775000	-4.910742000	1.441291000

1	1.669785000	-5.051828000	-0.132014000
1	3.869257000	-0.238077000	3.507536000
1	4.507225000	-1.373922000	2.271533000
1	4.647144000	0.385126000	2.015112000
1	4.977082000	2.766705000	-1.105275000
1	4.351936000	1.388569000	-2.070501000
1	3.959640000	3.069494000	-2.555707000
1	-2.034173000	3.813492000	3.853516000
1	-3.117460000	3.894924000	2.423635000
1	-2.819331000	2.328338000	3.221494000

Int. 2

Total energy -1445.747914 a.u.

Number of imaginary frequencies 0

6	-2.279519000	-1.303373000	-2.202291000
7	-2.701362000	-1.713848000	-0.939330000
6	-1.600825000	-1.815201000	-0.120233000
7	-0.510608000	-1.518310000	-0.867225000
6	-0.927907000	-1.181143000	-2.137453000
6	-1.736751000	-2.193100000	1.224686000
6	-3.003846000	-2.452838000	1.693803000
6	-4.131984000	-2.329144000	0.836761000
6	-3.968474000	-1.954858000	-0.467975000
6	0.815179000	-1.317056000	-0.350982000
6	1.260733000	-0.040070000	-0.156436000

6	2.751920000	0.159072000	0.046313000
8	2.994012000	0.862775000	1.164304000
6	4.371248000	1.215425000	1.387881000
6	1.542435000	-2.592298000	-0.152862000
8	2.526002000	-2.500083000	0.761504000
6	3.387944000	-3.646608000	0.878877000
8	1.240989000	-3.630415000	-0.720868000
8	3.602525000	-0.267154000	-0.703867000
1	-5.132600000	-2.518302000	1.208881000
1	-3.148628000	-2.735548000	2.731465000
1	-0.855727000	-2.241907000	1.853202000
1	-4.775037000	-1.822922000	-1.178395000
1	-0.216795000	-0.873188000	-2.888256000
1	-2.975460000	-1.126833000	-3.006404000
1	4.101996000	-3.389157000	1.661351000
1	2.810305000	-4.532400000	1.155507000
1	3.903706000	-3.825337000	-0.068383000
1	4.376007000	1.780163000	2.320643000
1	4.986371000	0.315693000	1.476239000
1	4.734493000	1.828389000	0.558808000
6	0.382302000	1.155881000	-0.168328000
6	-0.885254000	1.047368000	0.300339000
6	1.030280000	2.395736000	-0.690743000
6	-1.894753000	2.051018000	0.384485000
8	0.275432000	3.500432000	-0.536895000

8	2.130506000	2.419941000	-1.227547000
6	0.818728000	4.714247000	-1.075188000
1	0.060204000	5.475750000	-0.890002000
1	1.754932000	4.972287000	-0.571491000
1	1.005297000	4.609115000	-2.147786000
8	-1.953729000	2.674817000	1.602891000
8	-2.742894000	2.242983000	-0.493118000
6	-3.055965000	3.569189000	1.787823000
1	-2.950817000	3.952313000	2.805087000
1	-3.019069000	4.392114000	1.067119000
1	-4.011084000	3.045023000	1.676210000

TS3

Total energy -1445.743760 a.u.

Number of imaginary frequencies 1

6	-1.213903000	-0.434705000	-0.003187000
6	-0.900697000	1.818768000	-0.126709000
7	0.202752000	1.765100000	0.705534000
6	1.359291000	0.997099000	0.349285000
6	2.610558000	1.809439000	0.349356000
8	3.476692000	1.451576000	-0.612963000
6	1.245997000	-0.340095000	0.140569000
6	2.519101000	-1.120244000	-0.126432000
6	-0.045267000	-1.075620000	0.207731000
6	0.066459000	-2.531195000	0.552754000

8	-1.123987000	-3.158288000	0.582583000
6	-2.569759000	-0.895334000	0.078039000
7	-1.941794000	2.325905000	0.643584000
6	-0.927525000	2.053419000	-1.537585000
6	-2.096751000	2.483668000	-2.097101000
6	-3.241587000	2.761268000	-1.281172000
6	-3.135046000	2.696126000	0.077860000
6	-1.543655000	2.325179000	1.985253000
6	-0.237834000	1.972301000	2.012334000
8	2.795454000	2.725370000	1.130061000
8	3.461927000	-1.166782000	0.632145000
8	2.469351000	-1.706568000	-1.334551000
6	3.580118000	-2.565838000	-1.651941000
8	1.116407000	-3.101521000	0.804358000
8	-3.292033000	-0.717658000	1.058837000
8	-3.041516000	-1.389782000	-1.104387000
6	-4.438183000	-1.712311000	-1.128191000
6	4.772255000	2.081017000	-0.561251000
6	-1.094656000	-4.546009000	0.953048000
1	-4.182601000	3.061095000	-1.727780000
1	-2.164642000	2.620867000	-3.172118000
1	-0.046736000	1.815664000	-2.123359000
1	-3.931386000	2.945229000	0.768913000
1	0.443138000	1.876791000	2.843394000
1	-2.222776000	2.596201000	2.777886000

1	5.314850000	1.690890000	-1.422384000
1	4.669896000	3.167179000	-0.624324000
1	5.278724000	1.810705000	0.368970000
1	3.368572000	-2.954097000	-2.648705000
1	4.514774000	-1.998654000	-1.647220000
1	3.637238000	-3.376971000	-0.921464000
1	-2.137357000	-4.865042000	0.951491000
1	-0.512915000	-5.122492000	0.228235000
1	-0.655263000	-4.666760000	1.946946000
1	-4.630016000	-2.093691000	-2.132799000
1	-4.675116000	-2.473566000	-0.379072000
1	-5.046948000	-0.822772000	-0.935060000

Int. 3

Total energy -1445.774181 a.u.

Number of imaginary frequencies 0

6	-1.425099000	2.959246000	1.278002000
7	-2.085661000	2.070391000	0.404181000
6	-1.079811000	1.163453000	-0.222138000
7	0.188531000	1.687239000	0.399641000
6	-0.102100000	2.756296000	1.275363000
6	-1.073524000	1.318125000	-1.728532000
6	-2.134090000	1.835142000	-2.378188000
6	-3.273757000	2.382754000	-1.662132000
6	-3.187714000	2.534277000	-0.325670000

6	1.342694000	0.995208000	0.279052000
6	1.296821000	-0.351532000	-0.038385000
6	2.562412000	-1.047641000	-0.330267000
8	2.368490000	-2.254282000	-0.905292000
6	3.550520000	-3.042866000	-1.128012000
6	2.608269000	1.761152000	0.605388000
8	3.189799000	2.222548000	-0.506130000
6	4.463308000	2.878423000	-0.323064000
8	2.963771000	1.998693000	1.739791000
8	3.673195000	-0.588107000	-0.110714000
1	-4.158220000	2.712611000	-2.195917000
1	-2.130177000	1.879905000	-3.464779000
1	-0.197854000	0.945806000	-2.251404000
1	-3.939228000	3.042094000	0.272785000
1	0.675178000	3.210398000	1.870003000
1	-1.994337000	3.685370000	1.841293000
1	4.775679000	3.177857000	-1.323224000
1	4.353244000	3.748966000	0.328364000
1	5.174443000	2.172596000	0.112784000
1	3.196938000	-3.966929000	-1.586407000
1	4.237760000	-2.518012000	-1.796730000
1	4.051028000	-3.249119000	-0.178211000
6	0.016604000	-1.019975000	0.144130000
6	-1.153906000	-0.313717000	0.186973000
6	0.043602000	-2.496652000	0.477892000

6	-2.422609000	-0.978168000	0.579258000
8	-0.604148000	-3.228243000	-0.443119000
8	0.606746000	-2.945802000	1.451560000
6	-0.721027000	-4.631581000	-0.145802000
1	-1.244229000	-5.064502000	-0.999057000
1	0.268701000	-5.080991000	-0.028546000
1	-1.297212000	-4.766633000	0.773447000
8	-3.503506000	-0.401842000	0.012882000
8	-2.499682000	-1.951297000	1.308789000
6	-4.774010000	-0.984317000	0.348168000
1	-5.514717000	-0.377573000	-0.173844000
1	-4.817927000	-2.022873000	0.009139000
1	-4.935977000	-0.951471000	1.429184000

TS4

Total energy **-1445.725029 a.u.**

Number of imaginary frequencies **1**

6	-1.994402000	-2.592283000	-0.843031000
7	-2.229933000	-1.929344000	0.368695000
6	-1.151776000	-0.993119000	0.643064000
7	-0.196670000	-1.259122000	-0.550144000
6	-0.813065000	-2.249447000	-1.376046000
6	-0.698798000	-1.002282000	2.049759000
6	-1.503005000	-1.526584000	3.002974000
6	-2.756430000	-2.174151000	2.679333000

6	-3.047178000	-2.409503000	1.377860000
6	1.197890000	-0.934791000	-0.449297000
6	1.339564000	0.404337000	-0.092017000
6	2.672329000	1.084265000	0.080032000
8	2.964078000	1.242222000	1.383268000
6	4.167633000	1.983334000	1.660879000
6	2.175019000	-1.939598000	-0.814299000
8	3.426403000	-1.595082000	-0.428665000
6	4.479647000	-2.475271000	-0.852685000
8	1.920134000	-2.994183000	-1.390880000
8	3.378344000	1.432095000	-0.837842000
1	-3.422506000	-2.522899000	3.459586000
1	-1.195292000	-1.475334000	4.044148000
1	0.246619000	-0.527667000	2.283272000
1	-3.897718000	-2.992968000	1.039495000
1	-0.306429000	-2.609752000	-2.254653000
1	-2.718452000	-3.300085000	-1.222383000
1	5.398291000	-2.020394000	-0.480777000
1	4.341528000	-3.472675000	-0.426224000
1	4.502230000	-2.546516000	-1.943584000
1	4.240939000	2.016763000	2.748366000
1	5.034814000	1.476660000	1.228995000
1	4.083808000	2.990794000	1.245506000
6	0.123468000	1.103445000	-0.038140000
6	-1.032760000	0.233318000	-0.232524000

6	0.039480000	2.550095000	0.058417000
6	-2.163759000	0.647845000	-1.149475000
8	-1.255349000	2.990897000	0.018550000
8	0.985115000	3.313692000	0.188498000
6	-1.424260000	4.416511000	0.043469000
1	-2.502299000	4.581619000	0.008055000
1	-1.002322000	4.835888000	0.960880000
1	-0.936010000	4.876363000	-0.820433000
8	-3.338886000	0.678382000	-0.498973000
8	-2.015956000	0.896934000	-2.325014000
6	-4.468073000	1.133807000	-1.270439000
1	-5.312893000	1.116547000	-0.581861000
1	-4.286355000	2.147117000	-1.637850000
1	-4.641954000	0.466603000	-2.118933000

Pr

Total energy -1445.802244 a.u.

Number of imaginary frequencies 0

6	-0.115609000	0.892575000	-0.261584000
6	0.902751000	-0.125383000	0.240107000
7	0.088231000	-1.366975000	0.282549000
6	-1.266579000	-1.097005000	-0.026229000
6	-1.353195000	0.255379000	-0.362244000
6	0.722904000	-2.522581000	0.546540000
6	2.085388000	-2.598487000	0.367509000

7	2.723227000	-1.626149000	-0.403500000
6	2.105755000	-0.407828000	-0.640106000
6	3.912884000	-1.920338000	-1.034716000
6	4.481495000	-1.049430000	-1.929276000
6	3.827427000	0.154473000	-2.245103000
6	2.631973000	0.448752000	-1.599853000
1	5.421523000	-1.323268000	-2.397093000
1	4.258821000	0.852933000	-2.954133000
1	2.111603000	1.377853000	-1.785782000
1	4.330664000	-2.892271000	-0.803071000
6	-2.244972000	-2.150232000	-0.027576000
6	-2.599087000	0.940369000	-0.848489000
6	0.051761000	2.322041000	-0.331736000
6	1.436114000	0.199850000	1.682017000
1	0.116918000	-3.373739000	0.828771000
1	2.655453000	-3.489759000	0.583805000
8	-3.478945000	-1.717462000	-0.396226000
8	-2.015487000	-3.323370000	0.277442000
6	-4.509991000	-2.714085000	-0.423450000
1	-5.408946000	-2.186842000	-0.745399000
1	-4.258961000	-3.509264000	-1.131211000
1	-4.653336000	-3.148584000	0.570285000
8	-3.305413000	1.453257000	0.170200000
8	-2.909978000	1.008032000	-2.016842000
6	-4.468904000	2.213138000	-0.204875000

1	-4.907891000	2.549535000	0.734967000
1	-4.171601000	3.065257000	-0.821590000
1	-5.171789000	1.584744000	-0.758624000
8	1.318133000	2.713622000	0.036173000
8	-0.806222000	3.134501000	-0.658561000
6	1.556428000	4.128168000	0.040081000
1	2.590882000	4.245949000	0.366995000
1	1.416519000	4.548321000	-0.960574000
1	0.875456000	4.631127000	0.732711000
8	0.445093000	0.178916000	2.579084000
8	2.592981000	0.463154000	1.927728000
6	0.803763000	0.519641000	3.933551000
1	-0.123187000	0.446998000	4.502005000
1	1.550275000	-0.182369000	4.314333000
1	1.204252000	1.535944000	3.970870000
